# Oxidative Stress and Diminished Mitochondrial Proteostatic Reserve Are Linked to Enhanced mtUPR Initiation in Aged Mouse Muscle

**DOI:** 10.1111/acel.70573

**Published:** 2026-06-04

**Authors:** Grant R. Laskin, Baylah R. Mazonson, LaDora V. Thompson

**Affiliations:** ^1^ Department of Physical Therapy Boston University Boston Massachusetts USA

## Abstract

Mitochondrial dysfunction, impaired proteostasis, and reduced stress resistance and resilience are aging hallmarks. At the core of these hallmarks, the mitochondrial unfolded protein response (mtUPR) is a transcriptional pathway that restores mitochondrial proteostasis in response to proteotoxicity. Although the mtUPR is well studied in invertebrates and cell culture models, how the mtUPR is engaged in aged mammalian tissue is poorly defined. Here, we defined the extent to which repeated physical stress initiates mtUPR transcription in aged mouse skeletal muscle and assessed candidate regulatory mechanisms in vivo. Aged muscle exhibited reduced mitoprotective chaperone and protease availability and greater carbonylation of intermyofibrillar mitochondria relative to young muscle, suggesting diminished proteostatic reserve and increased oxidative burden. Short‐term physical stress induced a greater initiation of mtUPR genes in aged muscle than young muscle, coinciding with reduced physiological reserve. Physical stress shifted ATF5 localization from the mitochondria to the nucleus in the muscle of both ages, whereas *CHOP* mRNA and nuclear localization were selectively elevated in aged muscle. Mechanistically, we show mitochondrial reactive oxygen species (mtROS) contribute to mtUPR initiation in aged skeletal muscle. Using in vivo ChIP‐qPCR and in vitro knockdown/inhibition experiments, we provide support for CHOP as a redox‐sensitive factor contributing in part to the enhanced mtUPR initiation in aged mouse muscle, potentially linked to JNK signaling. Collectively, these data suggest reduced mitochondrial proteostatic reserve and mtROS signaling in aged muscle contribute to an amplified mtUPR transcriptional response following repetitive physical stress, providing the foundation to explore the mtUPR in mammalian aging.

## Graphical abstract

Aged mouse skeletal muscle exhibits greater mtUPR transcriptional initiation following repeated physical stress than young mice, coinciding with reduced mitochondrial proteostatic reserve and heightened oxidative burden. We implicate a mtROS‐JNK‐CHOP axis as a contributor to the enhanced response.

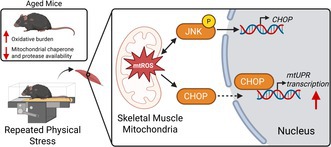

## 1 Introduction

Mitochondrial dysfunction and impaired proteostasis are two hallmarks of aging that contribute to functional decline (López‐Otín et al. 2023). Mitochondria rely on specialized proteostatic systems to maintain a functional pool, and age‐associated deterioration of these systems are major drivers of respiratory dysfunction (Srivastava 2017). Older organisms also exhibit impaired resistance (maintenance of function) and resilience (recovery of function) to stressors that challenge mitochondrial proteostasis (Laskin, Perazza, et al. 2025). Emerging evidence indicates the failure of aged mitochondria to appropriately remodel proteins following stress worsens functional impairments, whereas preserving mitochondrial proteostasis can improve healthspan (Drake and Yan 2017; Jensen and Jasper 2014). Thus, understanding mitochondrial proteostasis regulation (and its loss) in aging is likely to reveal causal mechanistic drivers underlying mitochondrial dysfunction and loss of functional independence.

The mitochondrial unfolded protein response (mtUPR) is a transcriptional pathway that restores mitochondrial proteostasis in response to stress by upregulating mitoprotective chaperones, proteases, antioxidants, and import machinery (Aldridge et al. 2007; Papa and Germain 2014; Sutandy et al. 2023). In invertebrates, mtUPR activation in response to mitochondrial stress preserves mitochondrial function, enhances healthspan, and promotes longevity (Houtkooper et al. 2013; Owusu‐Ansah et al. 2013). Despite the breadth of work in invertebrates, how mitochondrial stress engages the mtUPR in aged mammalian tissue remains poorly defined. Delineating the mtUPR in aged mammals is critical because it represents a key pathway intersecting established hallmarks of aging (e.g., mitochondrial dysfunction, impaired proteostasis), stress resilience, and functional preservation.

Recent work has demonstrated mtUPR initiation in young rodent muscle in response to a single acute bout of physical stress (Slavin et al. 2022; Turkel et al. 2025), but extension of these responses to aged mammalian muscle is limited. Whereas muscle transcriptional changes following a single bout of physical stress reflects an acute response to a novel challenge, transcriptional changes following repeated stress may better capture tissue resilience and reserve capacity, and thus be more relevant to age‐related functional decline (Cosarderelioglu et al. 2025; Egan and Zierath 2013). The aged muscle microenvironment also appears more susceptible to stress‐induced mtUPR activation than young muscle. Work in human cancer cells implicates mitochondrial reactive oxygen species (mtROS) in mtUPR activity, and oxidative stress is heightened in aged muscle and transiently increases with muscle use (Powers et al. 2011; Sutandy et al. 2023). Moreover, the canonical transcriptional mammalian mtUPR is largely attributed to the transcription factor ATF5 in cell models (Fiorese et al. 2016), with the integrated stress response (ISR) factors ATF4 and CHOP implicated as additional regulators (Horibe and Hoogenraad 2007; Sanfrancesco and Hood 2025). Notably, elevated ISR transcription factor activity is reported in aged muscle (Michel et al. 2025; Miller et al. 2023).

Despite these links, the extent to which repeated physical stress initiates the mtUPR transcriptional program in aged mammalian tissue is unclear. Additionally, although cell culture models have identified candidate regulatory mechanisms for stress‐induced mammalian mtUPR initiation, in vivo assessment of mtUPR regulation in aged mammalian tissue is lacking, and the aged tissue microenvironment may modify these pathways. Therefore, the purpose of this study was to define the extent to which repeated physical stress initiates the mtUPR in aged skeletal muscle and identify potential regulatory mechanisms in vivo.

Herein, we show repeated physical stress elicits a more pronounced initiation of the mtUPR transcriptional program in aged versus young skeletal muscle, and this response is linked to reduced availability of mitoprotective chaperones and proteases and increased intermyofibrillar (IMF) mitochondria carbonylation. Our data also show mtROS contributes to stress‐induced mtUPR initiation in aged muscle, and we implicate CHOP as a potential contributory transcription factor linking mitochondrial stress to enhanced mtUPR initiation in a redox‐sensitive manner, particularly in aged muscle. Overall, our findings suggest that reduced mitochondrial proteostatic reserve and heightened mtROS signaling in aged mammalian muscle may contribute to elicit a more pronounced mtUPR transcriptional program following repetitive stress. These results establish the necessary foundation to explore functional roles of the mtUPR in mammalian aging.

## 2 Results

### 2.1 Aging Reduces Mitoprotective Chaperone and Protease Stoichiometry in Skeletal Muscle

The mtUPR is initiated by an accumulation of unfolded or misfolded mitochondrial proteins that exceed the capacity for intramitochondrial repair. To estimate age‐dependent changes to intramitochondrial proteostatic capacity, we measured contents of mitoprotective chaperones and proteases in whole muscle lysates from young and aged mice of both sexes. We quantified both total chaperone and protease contents, and to approximate their availability relative to mitochondrial mass we quantified the stoichiometric ratio of each chaperone or protease to total OXPHOS proteins (e.g., a proxy of mitochondrial content) (Coen et al. 2015).

**FIGURE 1:**
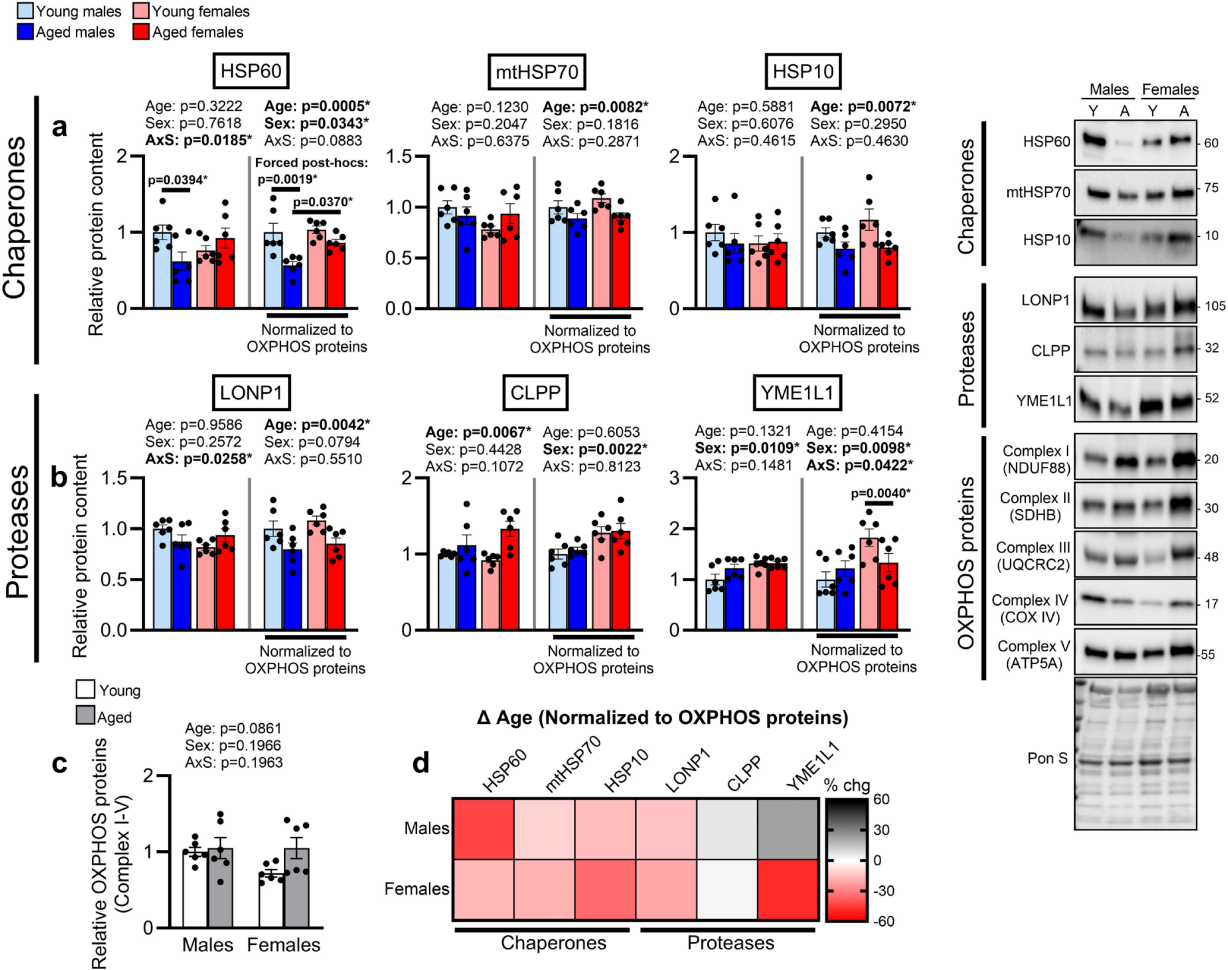
Aged skeletal muscle exhibits reduced availability of mitoprotective chaperones and proteases. The content of (a) mitochondrial chaperones HSP60, mtHSP70, and HSP10 and (b) proteases LONP1, CLPP, and YME1L1 were assessed in the medial gastrocnemius lysate of young (4‐months) and aged male (24‐months) and female (22‐months) mice (*n* = 6/group) by Western blot. Chaperones and proteases were analyzed as total contents (LEFT) or normalized to OXPHOS proteins as a surrogate marker of mitochondrial mass (RIGHT). (c) Total OXPHOS protein contents (I‐V) were assessed in the same lysates by Western blot. (d) Heat map visualizing the percent change between aged mice and their sex‐matched young counterparts in chaperone and protease contents normalized to OXPHOS proteins. (a–c) were analyzed by 2‐way ANOVA (Age × Sex) with Sidak's test used post hoc. Representative Western blot for (a–c) is shown on the right of the figure. Data are represented as mean ± SEM.

For total chaperone contents, we observed an Age × Sex interaction for HSP60, with post hoc tests showing lower HSP60 contents in aged relative to young males (Figure 1a). Total mtHSP70 and HSP10 did not differ by age or sex (Figure 1a). After normalizing chaperone contents to OXPHOS proteins, we detected main effect of Age for reduced stoichiometry of all three chaperones and for females to have a greater HSP60/OXPHOS protein ratio than males (Figure 1a). HSP60/OXPHOS proteins approached an Age × Sex interaction (*p* = 0.0883), with forced post hoc comparisons showing a lower HSP60/OXPHOS protein ratio in aged compared to young males and a greater ratio in aged females compared to aged males (Figure 1a).

For mitochondrial proteases, there was an Age × Sex interaction for total LONP1 contents, although pairwise post hoc comparisons were not significant (Figure 1b). There was a main effect of Age to reduce LONP1 contents relative to OXPHOS proteins (Figure 1b). There were main effects for total CLPP contents to be greater with age and total YME1L1 contents to be greater in females (Figure 1b). Both CLPP and YME1L1 showed a main effect for females to have a greater protease‐to‐OXPHOS protein ratio, with YME1L1 also showing Age × Sex interaction (Figure 1b). Post hoc testing revealed a reduced YME1L1/OXPHOS protein ratio in aged compared to young females (Figure 1b). Although the total average OXPHOS protein levels did not differ by age or sex, we did observe a larger variability of OXPHOS contents in aged mice (Figure 1c). There was a main effect of Age for greater Complex II (SDHB) contents when assessing the five OXPHOS subunits individually (Figure S1). Collectively, these observations show skeletal muscle aging reduces mitoprotective chaperone and protease abundance relative to a proxy of mitochondrial content (Figure 1d), suggesting that aged muscle mitochondria may have diminished resistance and resilience to proteotoxic stress.

### 2.2 Reduced Physiological Reserve in Aged Mice Coincides With Heightened mtUPR Transcriptional Initiation Following Repeated Physical Stress

**FIGURE 2:**
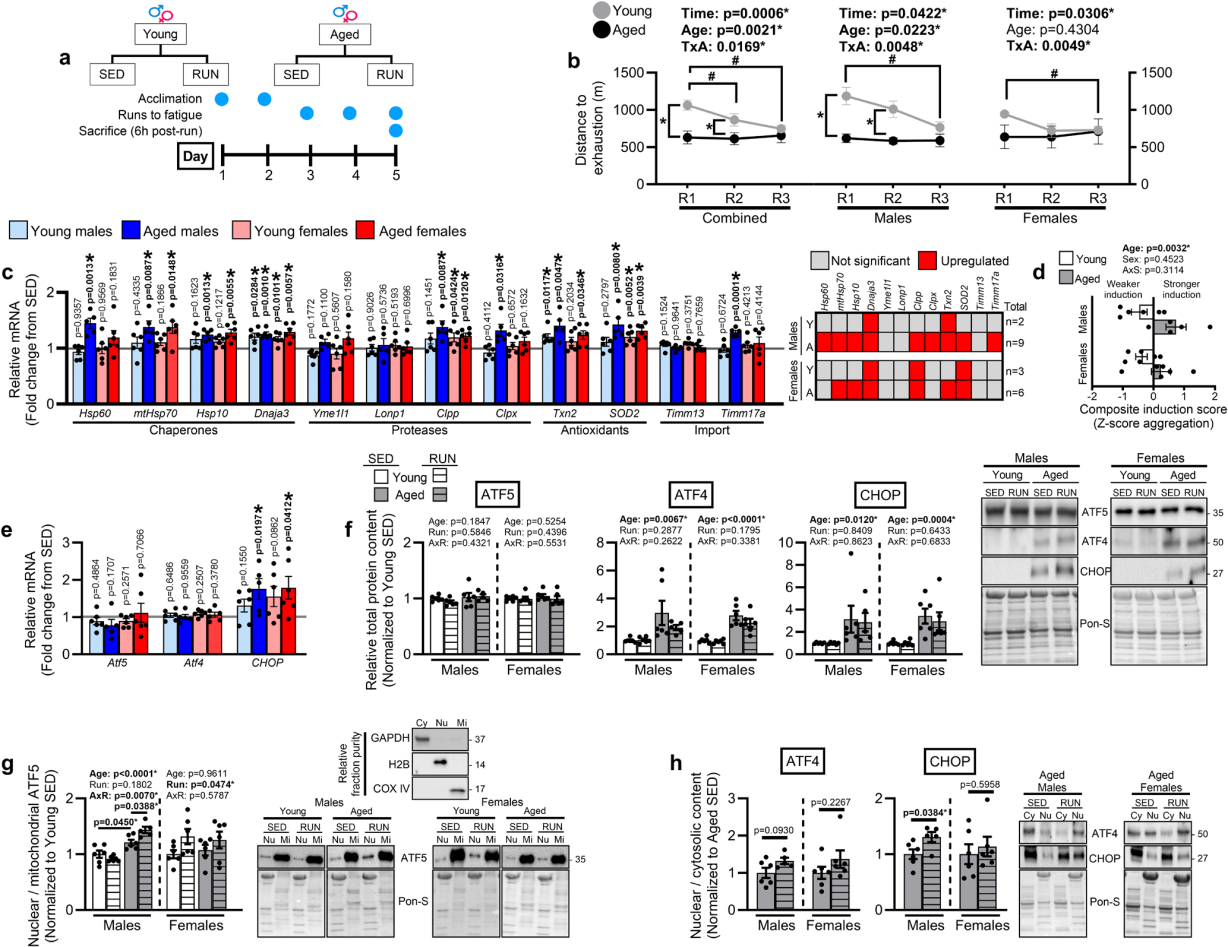
Repetitive physical stress induces a heightened initiation of the mtUPR transcriptional program in aged skeletal muscle. (a) Experimental groups and design for young (4‐months) and aged male (24‐months) and female (22‐months) mice assigned to remain sedentary (SED; *n* = 6/age/sex) or undergo 3 days of repetitive physical stress (RUN; *n* = 5 aged males, *n* = 6/age/sex all other groups). (b) Distance to exhaustion was calculated for each of the 3 consecutive runs to fatigue. The mRNA contents of (c) mtUPR target genes were assessed by qRT‐PCR in the medial gastrocnemius and (d) composite induction scores for each mouse were calculated using *z*‐score aggregation to assess the overall magnitude of mtUPR induction. (e) mtUPR transcription factor mRNAs were assessed by qRT‐PCR in the medial gastrocnemius. The total protein contents of (f) ATF5, ATF4, and CHOP were measured by Western blot in the medial gastrocnemius. The other gastrocnemius muscle was fractionated and the (g) mitochondrial‐to‐nuclear subcellular localization of ATF5 and (h) nuclear‐to‐cytosolic subcellular localization of ATF4 and CHOP were assessed by Western blot. Combined sexes, males only, and females only analyses in (b) were each assessed by 2‐way ANOVA (Time × Age) with Sidak's test used post hoc. #*p* < 0.05 vs. run 1 in young mice. *p < 0.05 between ages. Unpaired two‐tailed *t‐*tests were used to compare SED vs. RUN values in (c, e, h). (d) was analyzed by 2‐way ANOVA (Age × Sex). Males and females were each analyzed by 2‐way ANOVA (Age × Run) with Sidak's test used post hoc in (f, g). Representative Western blots for (f–h) are shown to the right of their respective bar graphs. Data are represented as mean ± SEM.

We hypothesized that the altered mitochondrial proteostasis signature observed in aged muscle would increase vulnerability to stress‐induced mitochondrial proteotoxicity, thereby promoting mtUPR transcriptional initiation. To test this, young and aged mice were subjected to a repetitive physical stress protocol consisting of 3 consecutive days of treadmill running to exhaustion to challenge muscle mitochondria (Figure 2a). Preliminary experiments showed reproducible declines in performance (e.g., reduced distance to exhaustion) by the third day of successive stress in young mice. We focused our primary assessment of mtUPR transcriptional initiation after the third consecutive run as this time point: (i) likely reduces transcriptional responses attributed to an acute, novel stress that may not reflect cumulative proteostatic stress (Egan and Zierath 2013), and (ii) provides a relevant window to detect mtUPR transcriptional responses to a repetitive stress paradigm capable of producing functional decline in young mice, allowing estimation of resilience to accumulated stress.

Functional capacity was assessed by measuring distance to exhaustion on each day. In combined‐sex analyses, there was a main effect for aging to reduce distance to exhaustion, a main effect of Time, and a Time × Age interaction (Figure 2b). Post hoc tests showed young mice ran shorter distances on runs 2 and 3 versus run 1, and aged mice ran shorter distances than young mice on runs 1 and 2 (Figure 2b). Sex‐stratified analysis in males recapitulated the statistical effects from combined‐sex analysis, with young males running less on run 3 versus run 1 and aged males performing worse than young males on runs 1 and 2 (Figure 2b). In females, there was a main effect of Time and Time × Age interaction, with post hoc testing revealing a decrease in distance between run 1 and run 3 in young females. The running distance of one aged female mouse approached outlier values (~2.5 SD from mean). In a separate analysis, excluding this animal's values revealed a main effect of Age to reduce distance to exhaustion and Time × Age interaction, with post hoc tests showing reduced distance in young females between run 1 and run 3 and lower distances in aged versus young females on runs 1 and 3 (Figure S2).

We then assessed the extent to which repetitive physical stress induces mtUPR transcription by evaluating putative mtUPR target genes cultivated from previous literature and the MitoCarta database (Aldridge et al. 2007; Papa and Germain 2014; Rath et al. 2021). These included chaperones (*Hsp60*, *mtHsp70*, *Hsp10*, *Dnaja3*), proteases (*Yme1l1*, *Lonp1*, *Clpp*, *Clpx*), antioxidants (*Txn2*, *SOD2*), and import machinery (*Timm13*, *Timm17a*). Physical stress induced changes to only a small number of genes in young mice, with young males showing elevated *Dnaja3* and *Txn2* mRNA and young females showing elevated *Dnaja3*, *Clpp*, and *SOD2* (Figure 2c). On the contrary, physical stress induced changes to a greater number of genes in aged muscle. Aged females showed significant elevations in 6 of the 12 genes analyzed, including the three genes altered in young females (*Dnaja3*, *Clpp*, and *SOD2*) as well as *mtHsp70*, *Hsp10*, and *Txn2* (Figure 2c). Aged males showed the greatest number of mtUPR genes altered by physical stress, including all the genes altered in aged females with the addition of *Hsp60*, *Clpx*, and *Timm17a* (Figure 2c). We estimated the collective magnitude of the physical stress‐induced mtUPR induction by calculating composite induction scores for each mouse based on z‐score aggregation of the log2FC relative to sedentary controls. All genes from the panel were included in the calculation except *Yme1l1*, *Lonp1*, and *Timm13* as they were unaltered in all groups. This assessment showed a main effect of aging for stronger induction of mtUPR genes (Figure 2d). Notably, including *Yme1l1*, *Lonp1*, and *Timm13* did not change results (data not shown).

In a separate cohort of young and aged males, we examined the response of the 9 altered mtUPR genes from Figure 2c to an acute, unaccustomed bout. All 9 genes responded in aged mice, whereas 6 of the genes (*Hsp60*, *mtHsp70*, *Clpx*, *Txn2*, *SOD2*, and *Timm17a*) were altered in young mice (Figure S3a). Calculating a composite induction score using either all 9 genes or by including only the 6 genes commonly altered in both ages showed that aged mice had a stronger induction of mtUPR genes in response to an acute bout compared to young mice (Figure S3b). Together, these data suggest physical stress elicits a greater mtUPR transcriptional response in aged muscle.

### 2.3 Repeated Physical Stress Alters ATF5 Subcellular Localization and Regulates CHOP in an Age‐Dependent Manner

ATF5 has emerged as a central mtUPR regulator in mammalian cells and young skeletal muscle (Fiorese et al. 2016; Slavin et al. 2022), and in vitro evidence implicates the transcription factors ATF4 and CHOP as additional contributors (Horibe and Hoogenraad 2007; Sanfrancesco and Hood 2025). Therefore, we evaluated these transcription factors following our physical stress in vivo. *CHOP* mRNA was elevated in response to physical stress in aged mice, whereas *Atf5* and *Atf4* mRNA contents were unaltered (Figure 2e). Despite elevated *CHOP* mRNA, total protein contents of ATF5, ATF4, and CHOP did not change with stress, although there were significant age‐related elevations of ATF4 and CHOP (Figure 2f). Notably, these proteins were not reliably detectable in young muscle lysates.

To estimate transcription factor activity, we assessed subcellular localization. ATF5 contains mitochondrial and nuclear localization signals, and translocates from the mitochondria to the nucleus during mitochondrial stress (Fiorese et al. 2016). In males, there was a main effect of Age and Age × Run interaction for ATF5 nuclear‐to‐mitochondrial localization (Figure 2g). Post hoc testing showed greater nuclear‐to‐mitochondrial ATF5 localization in aged sedentary males compared to young sedentary males, with physical stress further elevating nuclear‐to‐mitochondrial ATF5 localization in aged males compared to their sedentary counterparts (Figure 2g). In females, there was a main effect of running to increase nuclear‐to‐mitochondrial ATF5 (Figure 2g). Because ATF4 and CHOP were not reliably detectable in young muscle lysates, their localization analyses were restricted to aged mice. ATF4 localization was not significantly altered by physical stress in aged mice, although aged males approached greater nuclear‐to‐cytosolic localization in response to stress (*p* = 0.0930; Figure 2h). Physical stress increased nuclear‐to‐cytosolic CHOP localization in aged males but not aged females (Figure 2h). We also evaluated the transcription factor HSF1 as it can induce mitochondrial chaperones independently of the canonical mtUPR and contributes to mtUPR initiation in cancer cells (Sutandy et al. 2023). However, nuclear HSF1 was undetectable, *Hsf1* mRNA was unaltered by stress, and paradoxically total HSF1 protein was lower in aged males (Figure S4a–c), suggesting HSF1 is unlikely to be involved in our model. Our findings show induction of muscle mtUPR genes coincides with ATF5 nuclear translocation in both young and aged muscle, whereas *CHOP* transcription and nuclear translocation are altered only in aged muscle.

### 2.4 Aged Intermyofibrillar Mitochondria Exhibit Elevated Protein Carbonylation

**FIGURE 3:**
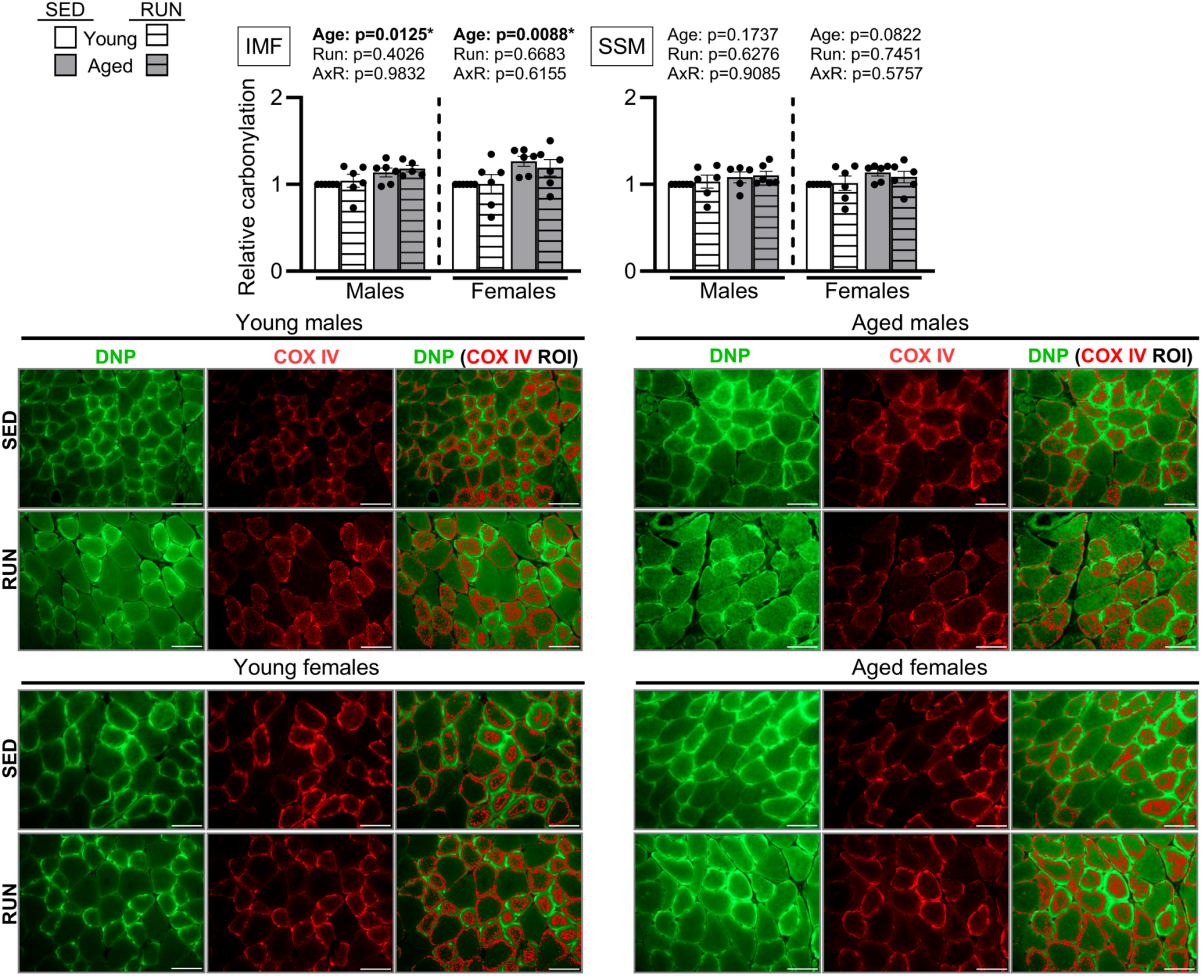
Aged intermyofibrillar mitochondria exhibit elevated protein carbonylation. Plantaris muscles of young (4‐months) and aged male (24‐months) and female (22‐months) mice subjected to treadmill stress (RUN; *n* = 5 aged males, *n* = 6/sex/age) or remained cage sedentary (SED; *n* = 6/sex/age) were assessed for subcellular carbonylation by fluorescent immunohistochemical labeling of the intermyofibrillar mitochondria (IMF) and subsarcolemmal mitochondria (SSM). Panels from left to right show fluorescent signal of DNP (carbonylation), COX IV (mitochondrial marker), and the co‐localization overlay of DNP^+^/COX IV^+^ regions of interest (ROIs). Images were taken at a 40× objective with a 50 μm scale bar superimposed. Males and females were each analyzed by 2‐way ANOVA (Age × Run). Data are represented as mean ± SEM.

We next sought to identify potential signals contributing to the enhanced mtUPR initation in aged muscle. Recent work in cancer cells implicates mtROS signaling as a driver of mtUPR initiation (Sutandy et al. 2023). Supporting this, skeletal muscle aging is associated with elevated oxidative stress, muscle mtROS are generated by physical stress, and dysfunctional mitochondria may overproduce mtROS (Bejma and Ji 1999; Powers et al. 2011). As direct assessment of mtROS in vivo is challenging due to their short half‐lives, we assessed protein carbonylation at muscle mitochondrial pools as an oxidative modification linked to mtROS production. In both sexes, there were main effect of Age for greater carbonylation at intermyofibrillar (IMF) mitochondria (Figure 3), the mitochondrial pool specialized for energy production directed towards contractile activity (Ferreira et al. 2010). Carbonylation of subsarcolemmal (SSM) mitochondria was unaltered with age, and physical stress did not further elevate carbonylation in either pool (Figure 3). Overall, these results indicate a selective elevation of oxidative damage in IMF mitochondria with age, consistent with the potential for increased mtROS production as a signal contributing to the augmented mtUPR in aged muscle.

### 2.5 Mitochondrial Redox Signaling Contributes to mtUPR Initiation in Myotubes and Aged Muscle

**FIGURE 4:**
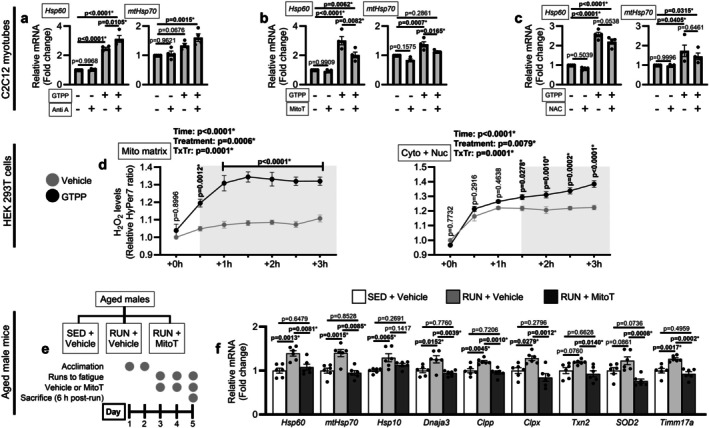
mtROS contributes to mtUPR initiation in myotubes and aged muscle. *Hsp60* and *mtHsp70* mRNAs were measured by qRT‐PCR in C2C12 myotubes (*n* = 4 biological replicates) treated with 10 μM Gamitrinib TPP (GTPP) for 3 h in the presence or absence of (a) 10 μM Antimycin A (Anti A) co‐treatment, (b) 1 h pre‐treatment 10 μM MitoTEMPO (MitoT) followed by co‐treatment, or (c) 1 h pre‐treatment 10 mM N‐acetylcysteine (NAC) followed by co‐treatment. (d) Spatial and temporal generation of ROS in response to 3 h GTPP treatment was assessed by capturing the fluorometric ratio at 30 min intervals from HEK293T cells (*n* = 4 biological replicates) transfected with H_2_O_2_‐sensitive probes targeted to the mitochondrial matrix (pCS2 + MLS‐HyPer7) or the cytosolic and nuclear compartments (pCS2 + HyPer7) using a plate reader. (e) Experimental groups and design for aged male mice (24‐mo) assigned to remain cage sedentary (SED) and receive vehicle treatment or undergo 3 days of physical stress (RUN) while receiving vehicle or 1 mg/kg MitoTEMPO (MitoT) 1 h prior to each run (*n* = 6/group). (f) mRNA contents of mtUPR target genes shown to be induced in Figure 2C were measured in the medial gastrocnemius of the mice by qRT‐PCR. (a–c, f) were analyzed by one‐way ANOVA with Tukey's test post hoc. (d) was analyzed by 2‐way ANOVA (Time × Treatment) with Sidak's test post hoc. Data are represented as mean ± SEM.

We next tested whether muscle mitochondrial redox signaling contributes to mtUPR initiation as reported in mammalian cancer cells (Sutandy et al. 2023). To accomplish this, we first assessed whether muscle mtROS was sufficient to initiate mtUPR transcription alone or required the presence underlying mitochondrial proteotoxicity using C2C12 myotubes. To induce mitochondrial matrix protein misfolding, myotubes were treated with the mitochondrial targeted HSP90 inhibitor Gamitrinib TPP (GTPP) (Sutandy et al. 2023). GTPP treatment occurred in the presence or absence of a mtROS inducer Antimycin A (Anti A) or scavengers MitoTEMPO (mitochondrial targeted) or N‐acetylcysteine (NAC). We focused on *Hsp60* and *mtHsp70* inductions as they are frequently measured markers of mtUPR initiation in vitro (Fiorese et al. 2016; Sutandy et al. 2023). GTPP alone induced *Hsp60* and *mtHsp70*, whereas Anti A or scavenger treatment alone did not have an effect (Figure 4a–c). GTPP+Anti A co‐treatment further elevated *Hsp60* and *mtHsp70* mRNA, while GTPP+MitoTEMPO partially reduced their inductions (Figure 4a,b). Substitution of MitoTEMPO with NAC, a primarily cytosolic antioxidant, only tended to attenuate *Hsp60* induction by GTPP and did not affect *mtHsp70* induction (Figure 4c).

As GTPP initiated the mtUPR without the presence of mtROS inducers, we quantified whether GTPP‐induced mitochondrial matrix misfolding generates mtROS directly in a non‐cancerous mammalian cell line. To accomplish this, we transfected HEK293T cells with HyPer7 plasmids encoding fully reversible H_2_O_2_‐sensitive probes targeted to the mitochondrial matrix or cytosolic/nuclear compartments (Pak et al. 2020). We attempted to transfect C2C12 myoblasts (data not shown); however, due to lower probe expression we used HEK293T cells to enable plate reader detection. GTPP treatment rapidly elevated H_2_O_2_ levels in the mitochondrial matrix (+0.5 h) with sustained elevation over the 3 h period (Figure 4d). Cytosolic/nuclear ROS increased at a later point (+1.5 h) and continued to rise until the 3 h mark (Figure 4d), suggesting mtROS efflux to the cytosol. Together, these findings show that in myotubes mtROS accompanies mitochondrial proteotoxicity and amplifies mtUPR transcriptional initiation when mitochondrial proteotoxicity is present, rather than being sufficient to drive the response alone.

We expanded upon our in vitro observations and asked whether reducing mtROS during physical stress would attenuate mtUPR gene induction in vivo. As we observed the greatest number of altered mtUPR genes and potentially larger alterations in regulatory transcription factor activity following physical stress in aged males than females (Figure 2c,g,h), we focused subsequent analyses on aged males. To test whether mtROS contributes to mtUPR transcriptional initiation in aged mammalian muscle, aged male mice underwent the physical stress protocol with or without MitoTEMPO administration (Figure 4e) and we reassessed the 9 mtUPR genes previously induced by physical stress (Figure 2c). Consistent with our previous cohort, all the genes were significantly elevated by physical stress, with the exceptions being the two antioxidant genes *Txn2* and *SOD2* which approached elevation (*p* = 0.0720 and *p* = 0.0861, respectively; Figure 4f). MitoTEMPO administration significantly attenuated the physical stress‐induced elevations of all mtUPR genes, besides *Hsp10* (Figure 4f). Notably, MitoTEMPO administration approached reducing *SOD2* mRNA lower than baseline sedentary values (*p* = 0.0736), consistent with MitoTEMPO's role as an SOD2 mimetic (Figure 4f). Importantly, the MitoTEMPO‐associated reductions in mtUPR genes were not attributable to differences in physical stress dose, as MitoTEMPO treatment did not alter distance to exhaustion and distances were comparable to our original cohort (Figure S5). Together with our in vitro data, these findings identify mtROS as a contributory factor to physical stress‐induced mtUPR transcriptional initiation in aged muscle.

### 2.6 CHOP Contributes to the Redox‐Sensitive Initiation of the Muscle mtUPR

**FIGURE 5:**
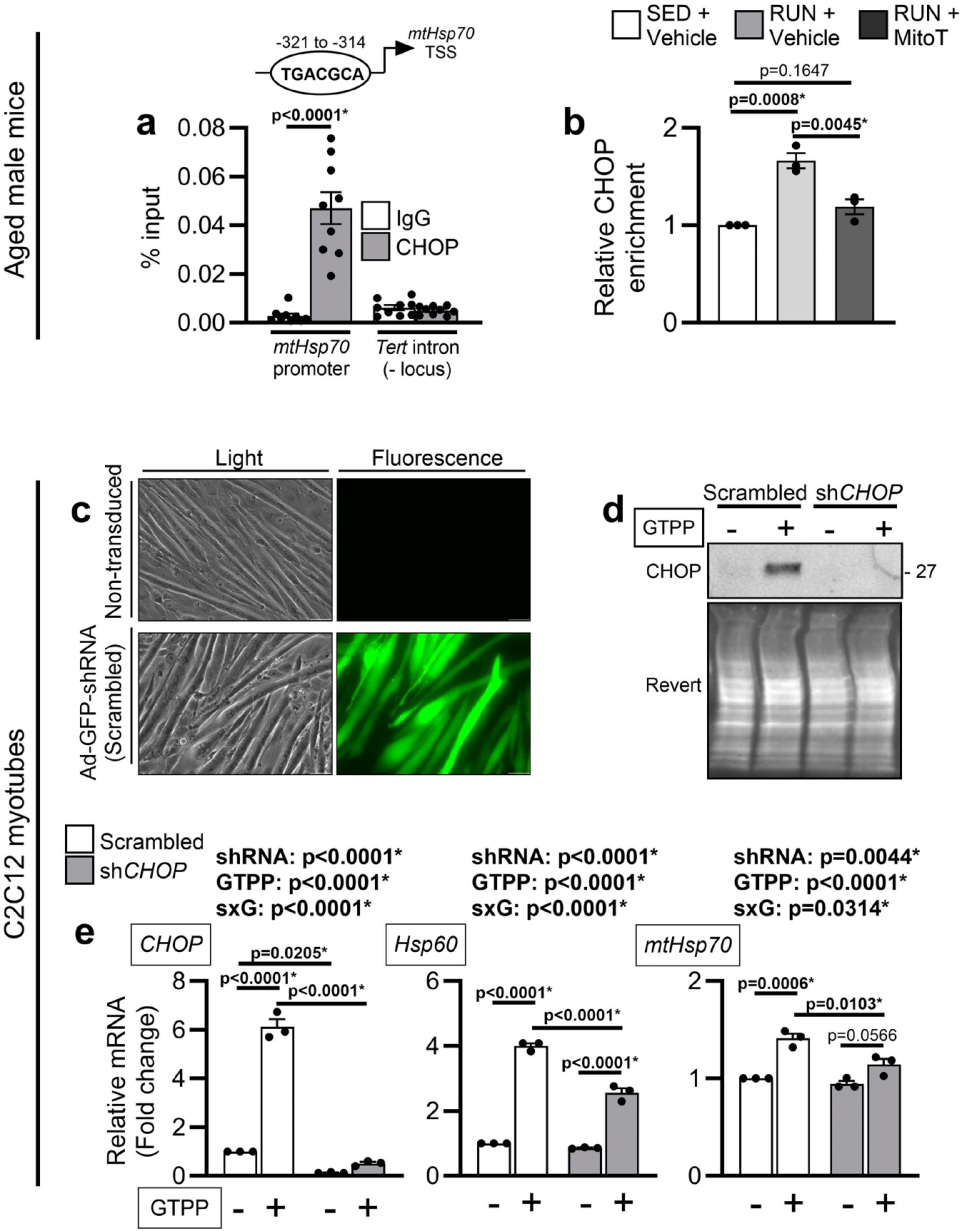
CHOP contributes to the redox‐sensitive initiation of the mtUPR in muscle. ChIP‐qPCR (*n* = 3 independent experiments) was performed on posterior limb muscles from aged male mice (24‐months) assigned to remain cage sedentary (SED) and receive vehicle treatment or undergo 3 days of physical stress (RUN) while receiving vehicle or 1 mg/kg MitoTEMPO (MitoT) 1 h prior to each run (*n* = 6/group). Muscles from two mice from the same group were pooled to yield *n* = 3 biological replicates/group. (a) ChIP‐qPCR to confirm CHOP occupancy at the *mtHsp70* promoter relative to IgG was performed without group stratification and analyzed by unpaired two‐tailed *t‐*test. (b) Corrected fold enrichments were calculated relative to the sedentary group sample within each ChIP replicate to evaluate treatment effects in promoter enrichment and analyzed by one‐way ANOVA with Tukey's test used post hoc. C2C12 myotubes transduced with adenoviruses encoding shRNA against *CHOP* or a scrambled control were treated with 10 μM Gamitrinib TPP (GTPP) or vehicle for 3 h. Transduction efficiency was validated by (c) fluorescent imaging of non‐transduced myotubes and myotubes transduced with scrambled control shRNA taken at a 20× objective with a 50 μm scale bar superimposed and by (d) CHOP protein levels by Western blot. (e) *CHOP*, *Hsp60*, and *mtHsp70* mRNAs were measured in the transduced myotubes by qRT‐PCR (*n* = 3 biological replicates) and analyzed by 2‐way ANOVA (shRNA × GTPP) with Sidak's test used post hoc. Data are represented as mean ± SEM.

*CHOP* mRNA was elevated by physical stress in aged muscle, CHOP protein was elevated in aged muscle, and CHOP localization shifted towards the nucleus following physical stress in aged males (Figure 2e,f,h). Since CHOP is redox‐sensitive (Yang et al. 2017) and in vivo links between CHOP and the mammalian mtUPR are limited, we assessed whether CHOP contributes to induction of mtUPR genes in aged muscle in a redox‐sensitive manner. Using skeletal muscle from the aged mice in the MitoTEMPO experiment (Figure 4e,f), we performed ChIP‐qPCR to identify CHOP occupancy at mtUPR promoters and assess its modulation by physical stress with or without MitoTEMPO administration. We focused on mapping potential CHOP binding sites within the *Hsp60* bidirectional promoter and the *mtHsp70* promoters as these showed the greatest inductions across our two separate experiments (Figures 2c and 4f). *In silico* analyses of proximal regions spanning −500 to +100 bp relative to transcription start sites revealed a CHOP::C/EBP‐like element harboring a TGCAAT core within the bidirectional *Hsp60* promoter (−118 bp) and a degenerate CHOP‐binding element with one mismatch to canonical CARE motifs within the *mtHsp70* promoter (−321 bp). Primer design for the *Hsp60* locus proved challenging due to a highly GC‐rich region, preventing efficient amplification despite multiple iterations; therefore, we focused on the *mtHsp70* locus for ChIP‐qPCR. We first analyzed samples independent of treatment group and confirmed CHOP enrichment at the *mtHsp70* promoter relative to IgG control (Figure 5a). By stratifying samples by treatment group, we observed physical stress increased relative CHOP occupancy at the *mtHsp70* locus, whereas MitoTEMPO administration attenuated this enrichment (Figure 5b).

To further validate the contribution of CHOP to mtUPR gene induction in muscle, we knocked down *CHOP* in C2C12 myotubes using adenoviral shRNA delivery and treated myotubes with GTPP. High transduction efficiency and specificity was confirmed as nearly all myotubes expressed the GFP reporter (Figure 5c), and sh*CHOP* abolished GTPP‐mediated CHOP protein and mRNA increases (Figure 5d,e). sh*CHOP* partially attenuated GTPP‐mediated elevations of *Hsp60* and *mtHsp70* mRNAs (Figure 5e). Together, the in vivo ChIP and myotube knockdown data support CHOP as a likely contributor to the redox‐sensitive initiation of the mtUPR in aged skeletal muscle.

### 2.7 JNK Is Hyperphosphorylated in Aged Muscle Following Physical Stress and Contributes to Redox‐Sensitive mtUPR Initiation in Myotubes

**FIGURE 6:**
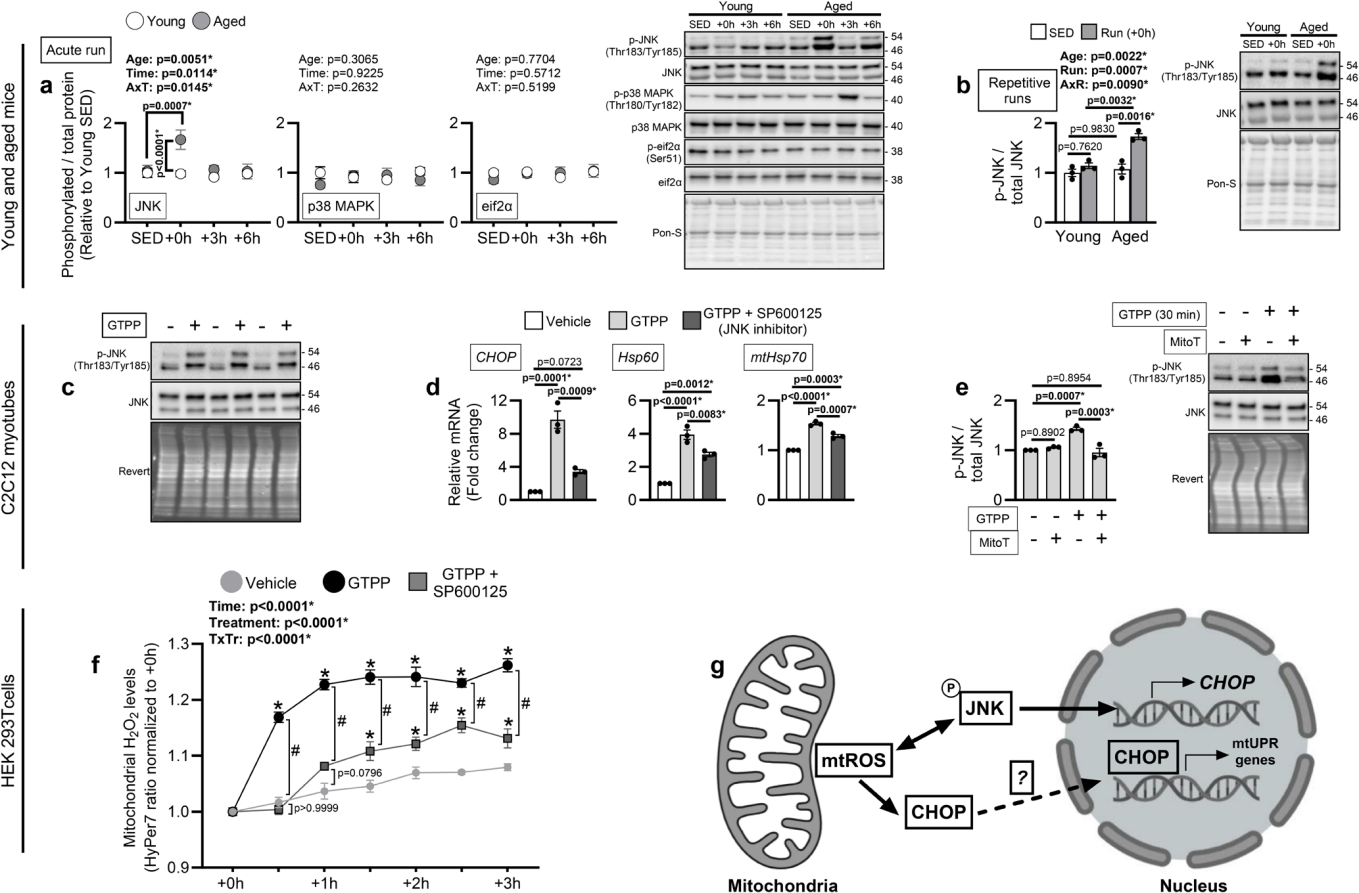
JNK hyperphosphorylation in aged muscle after physical stress is implicated in redox‐sensitive mtUPR initiation. (a) Young (4‐months) and aged (24‐months) male mice were randomly assigned to remain sedentary (SED) or undergo an acute run to exhaustion (*n* = 6/group) and were sacrificed at various time points post‐run (+0 h, +3 h, +6 h) to capture changes to JNK, p38 MAPK, and eif2α phosphorylation in the medial gastrocnemius. (b) JNK phosphorylation was measured in the medial gastrocnemius of young (5‐months) and aged (24‐months) male mice assigned to remain SED or undergo 3 consecutive days of running to exhaustion and sacrificed immediately post‐run (+0 h; *n* = 3/group). (c) JNK phosphorylation was confirmed in myotubes treated with 10 μM Gamitrinib TPP (GTPP) or vehicle for 3 h (*n* = 3 biological replicates). (d) *CHOP*, *Hsp60*, and *mtHsp70* mRNAs were assessed by qRT‐PCR in myotubes treated with 10 μM GTPP for 3 h in the presence or absence of 1 h pre‐treatment followed by co‐treatment of 25 μM SP600125 (*n* = 3 biological replicates). (e) JNK phosphorylation was assessed in myotubes treated with 10 μM GTPP for 30 min in the presence or absence of 1 h pre‐treatment followed by co‐treatment of 10 μM MitoTEMPO (MitoT) (*n* = 3 biological replicates). (f) mtROS generation in response to 3 h GTPP treatment in the presence or absence of 1 h pre‐treatment followed by co‐treatment of 25 μM SP600125 was assessed by capturing the fluorometric ratio at 30 min intervals from HEK293T cells (*n* = 4 biological replicates) transfected with H_2_O_2_‐sensitive probes targeted to the mitochondrial matrix (pCS2 + MLS‐HyPer7) using a plate reader. (g) Theoretical model in which mtROS generated from mitochondrial proteotoxic stress contributes to mtUPR initiation via CHOP. (TOP) mtROS induces JNK phosphorylation which contributes to *CHOP* induction. JNK activation reciprocally promotes mtROS generation. (BOTTOM) mtROS may also enhance CHOP occupancy at mtUPR promoters in response to stress, although the mechanism is undefined. This could potentially occur by stabilizing its nuclear localization or DNA interaction by activating redox‐sensitive dimerization partners. (a, b, f) were analyzed by 2‐way ANOVA (Age × Time, Age × Run, or Treatment × Time) with Sidak's test used post hoc. *p < 0.05 vs. vehicle and #*p* < 0.0001 GTPP vs. GTPP+SP600125 in (f). (d, e) were analyzed by one‐way ANOVA with Tukey's test used post hoc. Representative Western blots for (a, b, e) are shown to the right of their respective graphs. Data are represented as mean ± SEM.

Finally, we sought to identify signaling pathways contributing to the enhanced *CHOP* transcription observed in aged muscle following physical stress. We focused on upstream signaling pathways that are (i) redox‐sensitive, (ii) responsive to mitochondrial stress, (iii) able to induce *CHOP*, and (iv) reported to be activated by physical stress. Based on these criteria, we selected the kinases eif2α, JNK, and p38 MAPK as candidates (Baker et al. 2012; Bouviere et al. 2021; Horibe and Hoogenraad 2007; Park et al. 2015). Young and aged male mice were subjected to a single exhaustive run and sacrificed at multiple post‐run time points to capture changes in kinase phosphorylation. JNK phosphorylation showed main effects of Age, Time, and Age × Time interaction, with post hoc tests revealing JNK hyperphosphorylation immediately post‐run in aged but not young mice (Figure 6a). In contrast, eif2α and p38 MAPK phosphorylation were not changed (Figure 6a). We confirmed the age‐specific JNK hyperphosphorylation occurred in aged muscle following the 3‐day repetitive physical stress (Figure 6b).

Given the heightened JNK phosphorylation in aged males following physical stress, we investigated whether JNK contributes to *CHOP* transcription and mtUPR gene induction in a mtROS‐sensitive manner in vitro. After confirming GTPP treatment induces JNK phosphorylation in myotubes (Figure 6c), we subsequently treated myotubes with GTPP with or without SP600125, a JNK inhibitor. JNK inhibition markedly reduced GTPP‐mediated induction of *CHOP* mRNA and blunted induction of *Hsp60* and *mtHsp70* mRNAs (Figure 6d). To determine whether mtROS generated during mitochondrial proteotoxic stress contributes to JNK phosphorylation, we treated myotubes with GTPP for 30 min in the presence or absence of MitoTEMPO. MitoTEMPO abolished GTPP‐mediated JNK phosphorylation (Figure 6e). In other models, JNK shows reciprocal dependency with mtROS, whereby mtROS activates JNK and JNK activation promotes mtROS generation (Heslop et al. 2020). To determine whether JNK promotes mtROS generation in response to mitochondrial proteotoxicity, we reanalyzed mtROS in GTPP‐treated HEK293T cells expressing pCS2 + MLS‐HyPer7 with or without SP600125. Accordingly, JNK inhibition partially reduced GTPP‐induced mtROS generation (Figure 6f). These findings provide evidence that heightened stress‐induced JNK signaling in aged muscle may reflect a potential upstream redox‐sensitive node linking mtROS generation to amplified mtUPR initiation, in part by enhanced *CHOP* transcription (Figure 6g).

## 3 Discussion

The mtUPR is a mitoprotective transcriptional program linked to mitochondrial health in invertebrates but remains poorly defined in mammalian aging. Here, we sought to better understand the aged mammalian mtUPR by determining the extent to which repetitive physical stress initiates the mtUPR transcriptional program and by identifying key regulatory features in vivo. We show repeated physical stress elicits a more pronounced initiation of the mtUPR transcriptional program in aged versus young skeletal muscle. This age‐dependent transcriptional response coincided with reduced availability of mitoprotective proteostatic machinery and increased IMF carbonylation, suggesting an environment of impaired mitochondrial proteostasis and heightened oxidative burden. Mechanistically, our data indicates mtROS contribute to stress‐induced mtUPR initiation in aged muscle, and we show in cultured myotubes that mtROS contributes to mtUPR initiation only when mitochondrial proteotoxic stress is present but is insufficient on its own, consistent with our in vivo observations. Using in vivo ChIP‐qPCR and in vitro knockdown, we identify CHOP as a likely transcription factor contributing to induction of mtUPR genes following physical stress in a redox‐sensitive manner, particularly in aged muscle. Enhanced *CHOP* transcription following physical stress was associated with hyperphosphorylated JNK in aged muscle, and we provide evidence in myotubes that JNK may function as a potential mtROS‐sensitive regulator that regulates *CHOP*, amplifies mtROS, and thus promotes mtUPR gene transcription in response to mitochondrial proteotoxicity. Collectively, these findings support a model in which dysregulated mitochondrial proteostasis and mtROS signaling in aged mammalian muscle converge to elicit a more pronounced stress‐induced mtUPR transcriptional program.

The enhanced stress‐induced mtUPR transcriptional response in aged muscle coincided with lower mitochondrial chaperone and protease contents relative to total OXPHOS protein content, a proxy of mitochondrial content. Decreased mitoprotective stoichiometry may reflect reduced intramitochondrial protein repair capacity per unit mitochondrial mass, suggesting aged mitochondria exhibit a diminished proteostatic reserve and are therefore potentially closer to a threshold for proteotoxic stress. Further support of heightened susceptibility to mtUPR initiation in aged muscle is supported by evidence of an altered redox environment in aged mitochondria. In myotubes, we determined that mtROS contributes to mtUPR gene expression in the presence of mitochondrial proteotoxicity. Accordingly, in addition to reduced chaperone and protease stoichiometry we observed selective carbonylation of IMF mitochondria in aged muscle. Because IMF mitochondria are directly engaged during contractile activity (Ferreira et al. 2010), this pattern implies a chronic, age‐associated increase in mtROS exposure within the IMF pool. In support, previous work in rat hearts reported only IMF mitochondria produce significantly higher mtROS with aging, with no apparent differences between IMF and SSM in young rats (Suh et al. 2003). Although enhanced contractile activity during physical stress is known to produce mtROS (Powers et al. 2011), particularly in aged mitochondria (Bejma and Ji 1999), we did not observe additional IMF carbonylation 6 h post‐physical stress. However, as carbonylation is a cumulative and relatively stable marker of oxidative damage, our measure could integrate damage over time and may lack sensitivity to detect transient increases above already elevated baseline levels in aged tissue. As our tissue sampling time point (6 h post‐run) was selected to coincide with mtUPR transcriptional events (Turkel et al. 2025), we were limited to ex vivo measurement of oxidative damage products. Nonetheless, it is still likely physical stress induced transient mtROS generation as MitoTEMPO administration antagonized stress‐induced mtUPR gene induction. Therefore, elevated mitochondrial oxidative burden together with reduced proteostatic reserves likely produces an environment predisposing aged mouse muscle to more pronounced stress‐induced mtUPR initiation.

Intriguingly, the pronounced mtUPR signature exhibited in aged mice was associated with an age‐dependent performance trajectory. Our stress paradigm was based on pilot data showing reproducible performance decline in young mice after 3 days. In young animals we observed the expected decline, whereas aged animals started at a lower baseline and did not decline further with repeated stress. However, the performance pattern in aged mice conceptually aligns with loss of physiological reserve and narrowing of homeostasis range (homeostenosis) (Taffet 2023). Comparable age‐specific responses to repeated muscular performance are reported, as meta‐analyses show older human muscle exhibits less force loss following repetitive muscle contractions despite lower baseline strength than younger counterparts (Christie et al. 2011). The progressive performance decrease in young mice alongside a reduced capacity to alter mtUPR genes after three successive stressors suggests factors besides mitochondrial proteostasis are more likely to contribute to the performance decline in young animals. Conversely, the enhanced mtUPR induction in aged mice following the repetitive stress alongside reduced proteostatic reserves and greater oxidative burden plausibly reflects a diminished baseline of mitochondrial proteostatic control and thus increased vulnerability to stress‐induced mitochondrial protein misfolding, rather than an enhanced adaptive potential.

Our data suggest ATF5 may mediate a central mtUPR transcriptional arm in both ages, whereas CHOP emerges as an additional redox‐sensitive arm, particularly in aged muscle. We observed increased nuclear‐to‐mitochondrial ATF5 contents with physical stress in young and aged muscle, consistent with its role as a primary mammalian mtUPR regulator that translocates from mitochondria to the nucleus in response to proteotoxic stress (Fiorese et al. 2016). By contrast, *CHOP* transcriptional induction occurred following physical stress only in aged muscle. Although CHOP levels are primarily regulated transcriptionally (Yang et al. 2017), we did not detect greater CHOP protein contents following physical stress in aged muscle despite greater *CHOP* induction. However, stress‐induced proteins encoded from uORF‐containing transcripts (e.g., ATF4 and CHOP) are very low abundance in unstressed tissue and pose challenges to measure in whole muscle lysates (Miller et al. 2023); thus, we may have been limited by sensitivity to detect a modest accumulation of newly translated CHOP following physical stress in aged muscle when basal contents were already elevated. One candidate mechanism contributing to greater *CHOP* transcription via mtROS‐sensitive signaling is JNK. Indeed, we observed that physical stress induces JNK hyperphosphorylation in aged muscle, and in myotubes we place JNK as an mtROS effector upstream of *CHOP* and mtUPR gene transcription in response to mitochondrial protein misfolding. These findings are consistent with studies showing that JNK promotes ROS‐induced *CHOP* transcription (Chen et al. 2015) and regulates mtUPR‐inducible protease promoter activity (Horibe and Hoogenraad 2007). Our findings further suggest that JNK promotes mtROS production following mitochondrial protein misfolding, consistent with reports of mtROS‐JNK reciprocal dependency (Heslop et al. 2020). Together, these results identify JNK as a potential contributor to redox‐sensitive mtUPR initiation that warrants further investigation in aged mammalian tissue in vivo.

Our data also support a functional contribution of CHOP to mtUPR gene induction in aged muscle and myotubes. ChIP‐qPCR in aged muscle demonstrated CHOP enrichment at the *mtHsp70* promoter, with CHOP occupancy increasing with physical stress and being attenuated by MitoTEMPO, suggesting a redox‐sensitive role. In myotubes, *CHOP* knockdown abolished GTPP‐mediated CHOP induction and partially reduced mtUPR gene expression. Interestingly, despite increased *CHOP* transcription, elevated total CHOP levels, and nuclear CHOP content in aged muscle of both sexes, stress‐induced shifts in CHOP localization were restricted to aged males. Because CHOP regulates gene expression by heterodimerization (Yang et al. 2017), the sex‐specific shift in localization may suggest that additional stress‐responsive transcription factors stabilize CHOP nuclear localization in aged males, potentially in an mtROS‐sensitive manner (Figure 6e, BOTTOM). Although in vivo mtROS were not directly measured in our study, this hypothesis is consistent with a recent meta‐analysis demonstrating higher skeletal muscle mtROS production in males than females, with the sex difference becoming more pronounced with age (Junker et al. 2022). Therefore, greater stress‐induced localization shifts of CHOP in aged males could be due in part to greater mtROS signaling. Collectively, one explanation for our results is that ATF5 may function as a core mtUPR factor in mammalian muscle of both ages, whereas CHOP contributes to modulate the amplitude and/or composition of the mtUPR transcriptional response to a greater extent in aged mammalian muscle, in part due to the mitochondrial redox environment.

Recent work indicates that mtUPR transcription is orchestrated by multiple transcription factors that can act independently, redundantly, or cooperatively. For example, young ATF5‐KO mice exhibit compensatory increases in skeletal muscle CHOP content (Slavin et al. 2022), and analyses in HeLa cells show distinct fractions of the mtUPR transcriptome are regulated by CHOP independently, ATF5 independently, or CHOP+ATF5 in either cooperative or redundant manners (Uoselis et al. 2023). Consistent with these reports, *CHOP* knockdown only partially prevented mtUPR gene induction in our myotube model, likely due in part to redundancies with ATF5‐mediated mtUPR transcription. Moreover, there are likely additional unidentified factors acting independently of ATF5 and CHOP to drive the mammalian muscle mtUPR, as young male mice showed inductions of a few mtUPR‐associated genes without detectable changes to ATF5 localization or CHOP following repetitive physical stress. Candidate mtUPR regulatory transcription factors may be those that bind the recently described Mitochondrial Unfolded Protein Response Element (MURE) motifs, regulatory elements on mtUPR‐responsive genes in mammalian cells whose binding factors remain unelucidated (Aldridge et al. 2007). Future studies are needed to identify these factors and elucidate how they integrate to coordinate the mtUPR in aged mammalian tissue.

We also underscore potential cell type‐ and context‐specific mtUPR regulatory features. Our hypothesis that mtROS mediate the skeletal muscle mtUPR was based in part by findings in HeLa cells (Sutandy et al. 2023). Intriguingly, that work identified mtROS‐dependent activation of HSF1 as a driver of mtUPR gene transcription and did not observe effects after *CHOP* ablation. By contrast, we did not detect nuclear HSF1 in aged skeletal muscle and saw effects with *CHOP* ablation. These discrepancies may reflect differences in stress‐inducible transcriptional networks between cancer‐derived and non‐cancerous cells (Murray et al. 2004). Moreover, most mechanistic studies investigating regulation of the mammalian mtUPR are performed in vitro, and our data suggest the aged microenvironment likely influences mtUPR regulation. Therefore, our data indicates that the drivers of mtUPR initiation may shift between aged skeletal muscle and other cell/tissue types, highlighting the importance to study mtUPR regulation directly in aging mammalian tissues.

The finding that repetitive physical stress elicits a heightened mtUPR in aged mammalian muscle raises important questions regarding its functional consequence. It remains unclear whether the stress‐induced mtUPR in aged mammalian muscle is primarily protective or may alternatively propagate functional decline under certain conditions. In *C. elegans* and *Drosophila*, genetically manipulating the mtUPR enhances lifespan and mitochondrial function (Houtkooper et al. 2013; Owusu‐Ansah et al. 2013), whereas functional studies in aged mammals are largely correlative, such as associations between augmented basal mtUPR and extended lifespan or mitochondrial stress resilience in long‐lived mouse strains (Ozkurede and Miller 2019). Conversely, mtUPR overactivation can inadvertently propagate deleterious mitochondrial genomes by altering mitochondrial dynamics (Lin et al. 2016). Since aged skeletal muscle accumulates heteroplasmic mtDNA (Wachsmuth et al. 2016), aberrant mtUPR activation could theoretically favor retention of dysfunctional mitochondria. Thus, the enhanced stress‐induced mtUPR initiation we observed in aged mammalian muscle could represent an attempt to restore proteostasis in the setting of reduced proteostatic reserve and elevated oxidative burden, a maladaptive process impairing mitochondrial quality control, or a mixture of both depending on context. This study provides a mechanistic framework for future work to determine the functional ramifications of enhanced mtUPR initiation in mammalian aging.

There are limitations that warrant consideration. We employed single stress paradigms to activate the mtUPR in vivo and in vitro, and mtUPR dynamics may differ under alternative stressors. Moreover, the in vivo physical stress relied on volitional performance‐based criteria to determine exhaustion, and thus behavioral factors could influence willingness to perform. The in vivo transcriptional data were collected 6 h post‐stress and may not capture early or later signaling events. Comprehensive temporal profiling of mtUPR initiation and resolution is needed. mtROS generation was inferred from oxidative damage markers, pharmacologic interventions, and HyPer‐based assays in HEK293T cells rather than direct in vivo assessment. Our approach in measuring chaperone and protease abundance relative to OXPHOS at the whole‐muscle level does not resolve proteostatic capacity between mitochondrial subpopulations (IMF and SSM), and future work should profile these subpopulations to determine whether aging differentially alters their proteostatic machinery. Finally, muscle‐specific *CHOP*, *ATF5*, and/or *JNK* loss‐ and gain‐of‐function studies are required to fully define the contribution(s) of these pathways to the aged mammalian mtUPR and functional outcomes in vivo. In summary, we better characterize the mammalian mtUPR in aged tissue by demonstrating heightened initiation of the mtUPR transcriptional response following physical stress and linking this response to a microenvironment of reduced proteostatic reserve and elevated oxidative burden. We also provide in vivo evidence of potential regulatory contributions by mtROS signaling and the transcription factors ATF5 and CHOP in aged mammalian muscle, supported by in vitro experimentation. Overall, these findings establish a basis for future studies to define the functional roles of the mtUPR in mammalian aging, a pathway representing a critical juncture connecting stress resistance and resilience, impaired proteostasis, and mitochondrial dysfunction in late life.

## 4 Methods

### 4.1 Animals

Young (4–5‐months) male and female C57BL/6 mice were obtained from Charles River Laboratories. Aged male (24‐months) and female (22‐months) C57BL/6 mice were obtained from Charles River, Jackson Laboratories, or the National Institute on Aging (NIA) rodent colony. The ages of the aged cohorts were selected to correspond to approximately 75% of the C57BL/6 median lifespan (Turturro et al. 1999). All animals were housed in a temperature‐controlled (25°C), 12‐h light/dark cycle environment within the university vivarium, with ad libitum access to standard rodent chow and water. Tissues were harvested under 2%–3% isoflurane anesthesia. Mice were euthanized by cardiac excision. All procedures were approved by the university Institutional Animal Care and Use Committee (IACUC).

### 4.2 Repetitive Physical Stress Protocol

Young and aged male and female mice were randomly assigned to either a sedentary group (SED; *n* = 5–6 per age/sex) or an exhaustive running group (RUN; *n* = 6 per age/sex) subjected to three consecutive days of physical stress. The physical stress protocol is described in Supplementary Methods. On each day, mice ran until physical exhaustion (e.g., failure to run despite manual prodding). To minimize metabolic variability, food was removed on the third day 1 h prior to the final run. Mice were euthanized 6 h following the final run to coincide with reported initiation of mtUPR transcriptional events (Turkel et al. 2025). One plantaris muscle was embedded in OCT compound using isopentane chilled in a liquid nitrogen for histological analysis. Remaining tissues were snap‐frozen in liquid nitrogen and stored at −80°C. Running was conducted at the same time of day (e.g., start of the light cycle) to minimize circadian variability. Distance to exhaustion was recorded for each session.

### 4.3 In Vivo MitoTEMPO Administration and Phosphorylation Time Course

Aged male mice were randomly assigned as SED or to two RUN groups and subjected to the previously described physical stress protocol (*n* = 6/group). One RUN group received intraperitoneal injections of 1 mg/kg MitoTEMPO 1 h prior to each run. The other RUN group and SED mice received an equal volume of sterile PBS as vehicle. For the phosphorylation time course, young and aged male mice were randomly assigned to SED (*n* = 6/age) or euthanized at defined time points (*n* = 6/age/time point) after a single exhaustive run.

### 4.4 Cell Culture and Treatments

HEK 293 T cells and C2C12 myoblasts were purchased from ATCC (#CRL‐3216, #CRL‐1772) and cultured subconfluently at 37°C in 5% CO_2_ in high glucose Dulbecco's modified Eagle's media (DMEM; #10‐017‐CV; Corning Life Sciences) containing 10% fetal bovine serum (FBS; #30‐2020; ATCC) and 1% penicillin–streptomycin (PS; #15140122; Thermo Fisher Scientific). C2C12 myoblasts were seeded onto plates precoated with a 2D type I rat tail collagen matrix (#5153‐1KIT; Advanced Biomatrix) as described (Laskin, Steiner, et al. 2025). Differentiation was initiated at full confluency by washing with D‐PBS and switching media to high glucose DMEM with 2% horse serum (#16050130; Thermo Fisher Scientific) and 1% PS. Cells were allowed to differentiate for 6 days prior to treatments. Reagents used were 10 μM GTPP (#HY‐102007A; Medchem Express), 10 μM MitoTEMPO (#HY‐112879; Medchem Express), 10 mM NAC (#A7250‐25G; Sigma‐Aldrich), 10 μM Antimycin A (#A8674‐25MG; Sigma‐Aldrich), and 25 μM SP600125 (#8177S; Cell signaling).

### 4.5 Spatially Resolved Live‐Cell ROS Measurement

H_2_O_2_‐sensitive HyPer7 probes targeted to the mitochondrial matrix (pCS2 + MLS‐HyPer7; #136470) or the cytosol/nucleus (pCS2 + HyPer7; #136466) were acquired from Addgene (gifts from Dr. Vsevolod Belousov) (Pak et al. 2020). Plasmids were amplified in competent DH5α *E. coli* strain and extracted using Omega Bio‐Tek E.Z.N.A Endo‐free Plasmid DNA Mini Kit II (#D6950). HEK 293 T cells were seeded in 24‐well black walled imaging plates (#P24‐1.5P; Cellvis) and transfected at 60%–70% confluency overnight with 500 ng DNA/well using Lipofectamine 3000 (#L3000001; Thermo Fisher Scientific) at a 1:3 DNA: reagent ratio in Opti‐MEM (#31985070; Thermo Fisher Scientific). The following day, cells were treated with GTPP or vehicle in recording media (#21063029; Thermo Fisher Scientific) supplemented with 10% FBS. Fluorometric ratios of the oxidized (excitation/emission: 488/525) to reduced probes (excitation/emission: 405/525) were analyzed immediately following treatment (time 0) and every 30 min thereafter on a BioTek Synergy H1 plate reader. Background signal from non‐transfected cell wells was subtracted on each plate. For JNK inhibition experiments, cells were pre‐treated with SP600125 for 1 h followed by GTPP co‐treatment. As SP600125 exhibits intrinsic fluorescence, signals were normalized to the +0 h time point (e.g., start of GTPP treatment) within each well to correct for compound/media background differences.

### 4.6 Adenoviral‐Mediated shRNA Gene Knockdown

C2C12 myotubes were transduced using adenoviruses (Vector Biolabs) as previously described (Laskin, Steiner, et al. 2025). Each vector consisted of a replication deficient (ΔE1/3) human adenovirus type 5 backbone encoding a shRNA under control of a U6 promoter and co‐expressed GFP reporter driven by a CMV promoter. Adenoviruses encoding shRNA targeting *CHOP* (Ad‐m‐DDIT3‐shRNA; #shADV‐256,899) or a scrambled non‐targeted shRNA sequence (Ad‐GFP‐U6‐shRNA; #1122) were added to DM at multiplicity of infection (MOI) of 300 at 2 days of differentiation. Cells were infected with viruses for 6 h, after which the cells were washed once, and media was replaced with fresh DM. Myotubes were allowed to differentiate for another 4 days prior to treatment with 10 μM GTPP or vehicle.

### 4.7 Protein Extraction and Western Blot Analysis

Protein extraction and Western blot analysis were performed as described (Laskin et al. 2024). Full details are provided in Supplementary Methods. Antibodies are listed in Table S1.

### 4.8 Subcellular Compartment Fractionation

Cytosolic, nuclear, and mitochondrial enriched compartments were fractioned from one gastrocnemius muscle by differential centrifugation. Procedures are detailed in Supplementary Methods. Relative fraction purity was confirmed by immunoblotting for compartment‐specific markers GAPDH (cytosol), Histone H2B (nucleus), and COX IV (mitochondria).

### 4.9 Immunohistochemical Fluorescent Labeling of Subcellular Carbonylation

Fluorescent labeling and subcellular carbonylation analysis was performed as described (Kostal et al. 2011). Full details of tissue processing, labeling, and image analysis are described in Supplementary Methods. Briefly, plantaris cryosections (10 μm) were derivatized and labeled with DNP and COX IV (mitochondrial marker). COX IV^+^ signals were thresholded and defined as regions of interest (ROIs). Subcellular regions were segmented based on spatial localization. Three non‐overlapping images were taken from each slide at a 40× objective. A no antibody/reaction control slide was included with each batch to calibrate background fluorescence. To control inter‐batch variability from derivatization, slides were processed in batches containing one representative sample from each group. Fluorescent values were normalized to the young sedentary sample within each batch. Image analysis was performed blinded on *n* = 50 fibers/mouse.

### 4.10 RNA Extraction, cDNA Synthesis, and qRT‐PCR

Procedures were performed as described (Laskin et al. 2024). Full details are provided in Supplementary Methods. SYBR green primer sequences and TaqMan probes are provided in Tables S2 and S3, respectively.

### 4.11 ChIP‐qPCR

Nuclei were isolated from posterior hindlimb muscles of mice described in In vivo *MitoTEMPO administration*. Muscles were pooled to generate *n* = 3 biological replicates per treatment group (*n* = 2–3 pooled mice/replicate). All mice were included. Each independent ChIP experiment (*n* = 3) contained one pooled sample per experimental group. Full details and primers are provided in Supplementary Methods.

### 4.12 Statistical Analysis

All statistical tests are reported in the corresponding figure legend. For in vivo data including young and aged mice of both sexes and a physical stress and sedentary group, analyses were conducted sex stratified using 2‐way ANOVAs (Age × Run or Age × Time). Two‐way ANOVAs were applied to all other experiments with two factors (Age × Sex, shRNA × GTPP, Treatment × Time, Age × Time). “Time” factor was treated as a repeated measure when present. When an interaction by 2‐way ANOVA was significant, Sidak's test was used post hoc. One‐way ANOVA with Tukey's post hoc test was used for comparisons across multiple treatment groups with a single factor. Unpaired two‐tailed *t*‐tests were used when only two independent groups were present. Composite induction scores were derived by standardizing the log2FC value from run mice into *z*‐scores to ensure equal weighting and calculating the arithmetic mean of those *z*‐scores as the individual mouse induction score (e.g., *z*‐score aggregation) (Foroutan et al. 2018). All experimental procedures were performed using at least two independent animal cohorts or three independent biological replicates for in vitro studies, with data pooled for final analysis. Significance was set at *p* < 0.05. Analyses were conducted using GraphPad Prism (version 10.5).

## Author Contributions

G.R.L. and L.V.T. conceived and designed the study. All authors contributed to study design. G.R.L. and B.R.M. performed data collection and analysis. All authors aided in data interpretation. G.R.L. drafted the manuscript. All authors edited and approved the manuscript.

## Funding

This work was supported by the National Institute on Aging, K07 AG072124. Hevolution Foundation, HF‐AGE‐003. Travis Roy Foundation.

## Conflicts of Interest

The authors declare no conflicts of interest.

## Supporting information


**Data S1:** acel70573‐sup‐0001‐Supinfo.docx.

**Figure S1:** Individual OXPHOS protein complex subunits were assessed in the medial gastrocnemius lysate of young (4‐months) and aged male (24‐months) and female (22‐months) mice (*n* = 6/group) by Western blot and analyzed by 2‐way ANOVA (Age × Sex). Data are represented as mean ± SEM.

**Figure S2:** Distance to exhaustion in young (*n* = 6) and aged (*n* = 5) female mice with one aged female mouse outlying value omitted was analyzed by 2‐way ANOVA (Time × Age) with Sidak's test used post hoc. #*p* < 0.05 vs. run 1 in young mice. *p < 0.05 between ages. Data are represented as mean ± SEM.

**Figure S3:** The mtUPR transcriptional response to an acute, unaccustomed bout in young (4‐months; *n* = 6/group) or aged (24‐months; *n* = 3/group) male mice assigned to remain sedentary (SED) or undergo 3 days of repetitive physical stress (RUN). (a) mtUPR target genes were assessed by qRT‐PCR in the medial gastrocnemius and (b) composite induction scores for each mouse were calculated using *z*‐score aggregation to assess the overall magnitude of mtUPR induction using (LEFT) all genes in the panel or (RIGHT) only genes commonly induced in both ages. All data were analyzed using unpaired two‐tailed *t*‐tests. Data are represented as mean ± SEM.

**Figure S4:** HSF1 does not appear to be involved in the mtUPR transcriptional response in aged skeletal muscle at the time points assessed. (a) HSF1 total protein content was measured in the medial gastrocnemius of young (4‐months) and aged male (24‐months) and female (22‐months) mice assigned to remain sedentary (SED; *n* = 6/age/sex) or undergo 3 days of physical stress (RUN; *n* = 5 aged males, *n* = 6/age/sex all other groups) by Western blot and assessed by 2‐way ANOVA (Age × Run). The other gastrocnemius of aged mice was fractionated and the (b) nuclear‐to‐cytosolic subcellular localization of HSF1 was measured by Western blot. (c) *Hsf1* mRNA content was measured in the medial gastrocnemius by qRT‐PCR and analyzed by unpaired two tailed *t*‐tests. Representative Western blot for (a) is shown to the right of the respective bar graph. Data are represented as mean ± SEM.

**Figure S5:** Distance to exhaustion was calculated for each of the 3 consecutive runs to fatigue in mice treated with vehicle or 1 mg/kg MitoTEMPO (MitoT) and analyzed by 2‐way ANOVA (Time × Treatment). Data are represented as mean ± SEM.

**Table S1:** Western blot antibodies.

**Table S2:** qRT‐PCR SYBR Green primer sequences.

**Table S3:** TaqMan primers.

## Data Availability

Data supporting the findings of this study are available from the corresponding author upon reasonable request.
