# Supplementary material for: Oxidative Stress and Diminished Mitochondrial Proteostatic Reserve Are Linked to Enhanced mtUPR Initiation in Aged Mouse Muscle
Source: Aging Cell. 2026 Jun 4;25(6):e70573. doi: 10.1111/acel.70573 (PMC13238549; doi:10.1111/acel.70573)
Supplement: Supplementary file 1 — Data S1: acel70573‐sup‐0001‐Supinfo.docx. [file ACEL-25-e70573-s002.docx]

*Repetitive physical stress protocol*

The protocol was adapted from a previous study (Triolo et al., 2022). Mice in the RUN group underwent two acclimation sessions, 48 and 24 h prior to the first experimental run. Each acclimation session involved 5 min of stationary treadmill exposure, followed by running at 5 m/min for 5 min and 10 m/min for 5 min on a 10% incline. Experimental sessions began with 5 m/min for 5 min, then 10 m/min for 10 min, followed by progressive speed increases of 1 m/min every 2 min until exhaustion, all at 10% incline.

*Total protein extraction and Western blot analysis*

~25 mg of the medial gastrocnemius were glass-on-glass homogenized (10 µL/mg muscle) or C2C12 myotubes were lysed in buffer consisting of 50 mM of HEPES (pH 7.4), 0.1% Triton X-100, 4 mM of EGTA, 10 mM of EDTA, 50 mM of Na_4_P_2_O_7_, 100 mM of β-glycerophosphate, 25 mM of NaF, 5 mM of Na_3_VO_4_, and 10 µL/mL of protease inhibitor cocktail (PIC; #P-8340; Sigma-Aldrich). Homogenates were centrifuged at 10000*g* for 10 min at 4°C, and the resulting supernatant was collected. Protein concentrations were determined using the Bradford assay. Samples were normalized to equal concentrations, diluted in 2× Laemmli buffer, and denatured by boiling at 95°C for 5 min.

Protein was separated using 4%-20% Mini-PROTEAN TGX precast protein gels (Bio-Rad Laboratories) and wet transferred to PVDF membranes. Transfer efficiency and uniform loading were verified using Ponceau-S or Revert 700 staining. Membranes were blocked with 5% nonfat dried milk diluted in Tris-buffered saline + 0.1% Tween-20 (TBST) and incubated overnight with primary antibodies diluted in TBST at 4°C. Protein-antibody complexes were visualized using Clarity ECL substrate (#1705061; Bio-Rad) on a ChemiDoc Touch imaging system. Pixel density for each target protein was normalized to the corresponding total protein stain prior to downstream analysis. Transcription factor localization analysis was performed by loading equal amounts of fractionated protein in side-by-side lanes followed by densitometric quantification of pixel intensity ratios. Densitometric analysis was performed using ImageLab (Bio-Rad) and ImageJ software (National Institutes of Health).

*Muscle subcellular compartment fractionation*

Procedures were modified from previous reports (Dias et al., 2020). A single gastrocnemius muscle was homogenized in 10 volumes (10 µL/mg muscle) of mitochondrial buffer consisting of 5 mM HEPES (pH 7.4), 1 mM EGTA, 220 mM mannitol, 70 mM sucrose, 5 mM Na_3_VO_4_, and 10 µL/mL protease inhibitor cocktail. The homogenate was centrifuged for 5 min at 2400 *g* at 4°C. The supernatant (S_1_; cytosolic and mitochondrial-enriched fractions) was transferred to a separate tube and the remaining nuclear-enriched pellet was retained on ice. To remove remaining nuclear debris, S_1_ was clarified twice by centrifugation at 2400 *g* at 4°C for 5 min. The clarified supernatant was then centrifuged for 25 min at 20000 *g* at 4°C to pellet crude mitochondria. The resulting supernatant (S_2_) was transferred into a new tube and the crude mitochondrial pellet was washed by fully resuspending in 200 µL of mitochondrial buffer. S_2_ was clarified and the mitochondrial enriched pellet was washed twice more after centrifugation at 25 min at 20000 *g* at 4°C. S_2_ was collected as the cytosolic-enriched fraction and the crude mitochondrial pellet was fully resuspended by vigorous pipetting in 65 µL in whole muscle lysis buffer and saved as the mitochondrial-enriched fraction. The nuclear pellet was washed three times in 800 µL of buffer consisting of 10 mM NaCl, 1.5 mM MgCl_2_, 20 mM HEPES (pH 7.4), 20% glycerol, 0.1% Triton X-100, 1 mM DTT, and 10 µL/mL PIC. Between each wash, samples were centrifuged at 2400 *g* for 5 min at 4°C and the supernatant discarded. After the final wash, the pellet was resuspended in 500 µL of whole muscle lysis buffer and homogenized using a glass-on-glass homogenization. Samples were centrifuged at 10000 *g* for 10 min at 4°C, and the supernatant was collected as the nuclear-enriched fraction. Samples were normalized to equal concentrations by Bradford assay, diluted in 2× Laemmli buffer, and denatured by boiling at 95°C for 5 min.

*Immunohistochemical fluorescent labeling of subcellular carbonylation*

**Processing and labeling:** Cryosections (10 µm) of plantaris muscle were rehydrated with TBS for 5 min then fixed for 10 min in 4% PFA in PBS. Slides were washed with TBS, permeabilized with TBS + 0.3% Triton X-100 for 10 min, then washed again. Derivatization was performed by incubating sections with 400 µL of 0.1% 2,4-dinitrophenylhydrazine (DNPH) in 2 M HCl in TBS for 1 h. The reaction was stabilized by removing the DNPH solution and incubating with 400 μL of 30 mM sodium cyanoborohydride in 0.1 M phosphate buffer (pH 6.0) for 1 h. Following additional washes, sections were blocked for 1 h in 5% normal serum diluted in TBS + 0.3% Triton X-100. Slides were incubated overnight at 4°C with conjugated primary antibodies against DNP (1:25 dilution; #A11097; Invitrogen) and COX4 (1:50 dilution; CL647-11242; ProteinTech) diluted in TBS + 1% BSA. The following day, slides were washed then sealed with antifade medium (#8961S; Cell signaling).

**Image-wide thresholding:** COX4 immunofluorescence images were processed in ImageJ (NIH). A freehand line was drawn through the cytoplasm of ten randomly selected cells per image, avoiding regions of obvious positive signal. The mean gray value of each region of interest (ROI) was recorded, and the threshold was calculated as the average of the 10 ROIs plus two standard deviations. Pixels exceeding this value were considered COX4^+^. Images were converted to 8-bit format, and the calculated threshold was applied to generate a binary mask of COX4^+^ signal.

**Identification of fiber mitochondrial regions:** For each cell, the cytoplasmic area was outlined on the carbonyl (CBN) channel and transposed to the COX4 channel. A macro was developed to semi-automate subsequent steps. This ROI was duplicated to create a cropped image, then concentrically scaled to 90% of its original size. The XOR function was used to define the outer 10% of the cell as the subsarcolemmal (SSL) region, while the inner area represented the interfibrillar (IF) region. COX4^+^ signal from the binary mask was intersected with the whole-cell, SSL, or IF ROIs to generate “AllMito,” “SSLMito,” and “IFMito” regions, respectively.

**Measurement of carbonyl fluorescence:** The whole-cell ROI from the COX4 channel was applied to the CBN image. Mean gray values were measured for each ROI (AllMito, SSLMito, IFMito) on the CBN channel to assess carbonylation in COX4^+^ regions. Cytosolic carrbonylation was assessed in the mitochondrial void cytosolic region by selecting the COX4^-^ regions using the XOR function.

*RNA extraction, cDNA synthesis, and qRT-PCR*

~20 mg of medial gastrocnemius muscle or C2C12 myotubes were homogenized in 500-600 µl of Zymo Tri Reagent. RNA was isolated using a Zymo Direct-zol RNA Miniprep kit (#R2050). RNA quantity and purity were determined spectrophotometrically by the 260/280 nm absorbance ratio. cDNA was synthesized from 1 µg of RNA using a High-Capacity cDNA Reverse Transcription Kit (#4368814; Thermo Fisher Scientific). qRT-PCR was conducted on QuantStudio5 (Thermo Fisher Scientific) using PowerUp SYBR Green Master Mix (#A25742; Thermo Fisher Scientific) or TaqMan Fast Advanced Master Mix (#4444557; Applied Biosystems). SYBR Green cycling conditions were an initial 2 min at 50°C and 2 min at 95°C, followed by 40 cycles with each cycle consisting of a 15 s denature step at 95°C, a 15 s annealing step at 55°C, and a 1 min extension step at 72°C. Melt curve analysis was performed for each primer pair to confirm specificity, and amplicon size was verified by 2% agarose gel electrophoresis prior to experimentation. TaqMan cycling conditions were an initial 2 min at 50°C and 10 min at 95°C, followed by 45 cycles with each cycle consisting of a 15 s denature step at 95°C, a 1 min annealing step at 60°C, and a 1 min extension step at 60°C. Relative expression levels of each target gene were normalized using the ΔΔC_t_ method using *Rplp0* as the reference gene.

*ChIP-qPCR*

Muscles were minced in 10 mM KCl, 10 mM HEPES-KOH (pH 7.3), 5 mM MgCl_2_, 0.1% Triton-X, and 10 µL/mL PIC and homogenized. Lysates were crosslinked with 1% formaldehyde for 10 min and quenched with 0.125 M glycine for 8 min. Nuclei were isolated as described (Joshi et al., 2017) and sonicated in 500 µL of buffer containing 10 mM EDTA, 1% SDS, 20 mM Tris-HCl (pH 8.0), 150 mM NaCl, 0.1% sodium deoxycholate, 1% Triton-X, and 10 µL/mL PIC using a Fisher 100 Sonic Dismembrator (50% amplitude; eight 1-min cycles, 1 s ON/1 s OFF, 1 min rest between cycles). Crosslinked chromatin was cleared by centrifugation at 12,000 *g* for 10 min at 4°C. Fragment size of 200-700 bp and DNA concentration were confirmed by high salt decrosslinking at 65°C overnight followed by 30 min of 0.1 mg/mL RNAse at 37°C and 2 h of 0.5 mg/mL Proteinase K at 56°C. DNA was purified with spin columns (#14209; Cell signaling). Ten µg chromatin was incubated overnight with rotation at 4°C with anti-CHOP (2 µg; #2895) or Mouse IgG2a antibodies (#61656) acquired from Cell signaling. ChIP was performed using reagents from the Cell signaling SimpleChIP Plus Sonication Chromatin IP kit (#56383). qPCR was performed with 2 µL DNA template using PowerUp SYBR Green Master Mix as described in *RNA extraction, cDNA synthesis, and qRT-PCR,* adjusting annealing temperature to 60°C. Percent input of CHOP IP was compared to IgG to confirm occupancy. A negative locus for CHOP occupancy (*Tert* intronic region) was included in each replicate. Corrected fold enrichment (IgG signal subtracted from percent input CHOP IP) relative to the sedentary group sample within each ChIP replicate was assessed to evaluate treatment effects in promoter enrichment. Primers used were: *mtHsp70* promoter (F: 5’-CCTCACACATGCGCTTCAC-3’; R: 5’-CCTTAGGACGGGAAAGACTCA-3’; Amplicon: 142) and *Tert* intron (F: 5’- CTAGCTCATGTGTCAAGACCCTCTT-3’; R: 5’-GCCAGCACGTTTCTCTCGTT-3’; Amplicon: 110).

**References**

Dias, P. R. F., Gandra, P. G., Brenzikofer, R., & Macedo, D. V. (2020). Subcellular fractionation of frozen skeletal muscle samples. *Biochemistry and Cell Biology = Biochimie Et Biologie Cellulaire*, *98*(2), 293–298. https://doi.org/10.1139/bcb-2019-0219

Joshi, S., Ueberschlag-Pitiot, V., Metzger, D., & Davidson, I. (2017). Improved Protocol for Chromatin Immunoprecipitation from Mouse Skeletal Muscle. *Journal of Visualized Experiments : JoVE*, (129), 56504. https://doi.org/10.3791/56504

Triolo, M., Oliveira, A. N., Kumari, R., & Hood, D. A. (2022). The influence of age, sex, and exercise on autophagy, mitophagy, and lysosome biogenesis in skeletal muscle. *Skeletal Muscle*, *12*(1), 13. https://doi.org/10.1186/s13395-022-00296-7
