# Supplementary figures and images for: Oxidative Stress and Diminished Mitochondrial Proteostatic Reserve Are Linked to Enhanced mtUPR Initiation in Aged Mouse Muscle
Source: Aging Cell. 2026 Jun 4;25(6):e70573. doi: 10.1111/acel.70573 (PMC13238549; doi:10.1111/acel.70573)

□ Young  
■ Aged

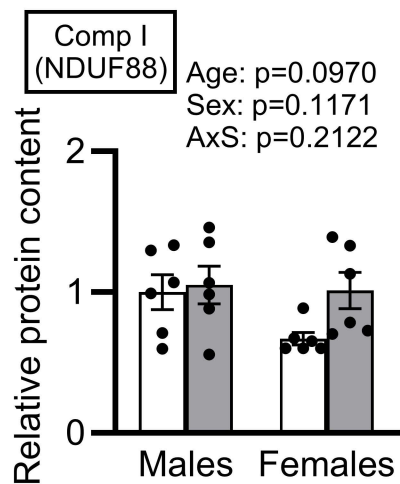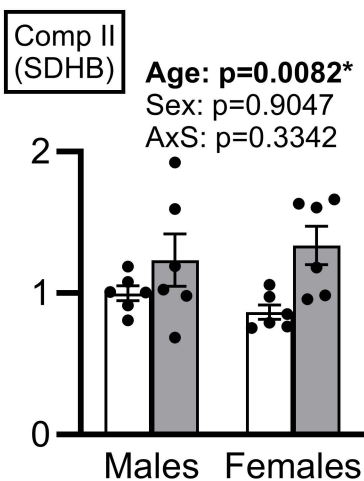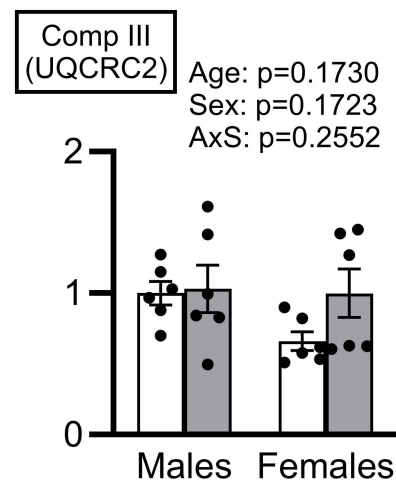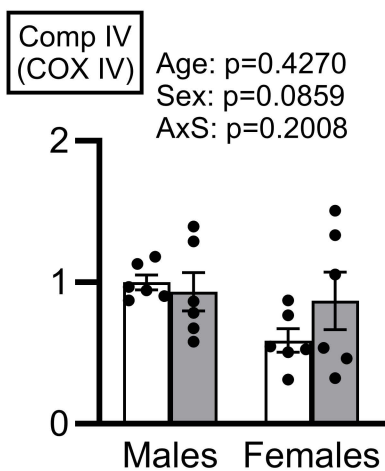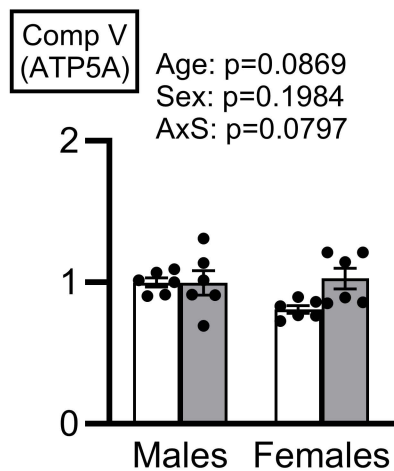

Supplement: Supplementary file 2 — Figure S1: Individual OXPHOS protein complex subunits were assessed in the medial gastrocnemius lysate of young (4‐months) and aged male (24‐months) and female (22‐months) mice (n = 6/group) by Western blot and analyzed by 2‐way ANOVA (Age × Sex). Data are represented as mean ± SEM. [file ACEL-25-e70573-s003.pdf]

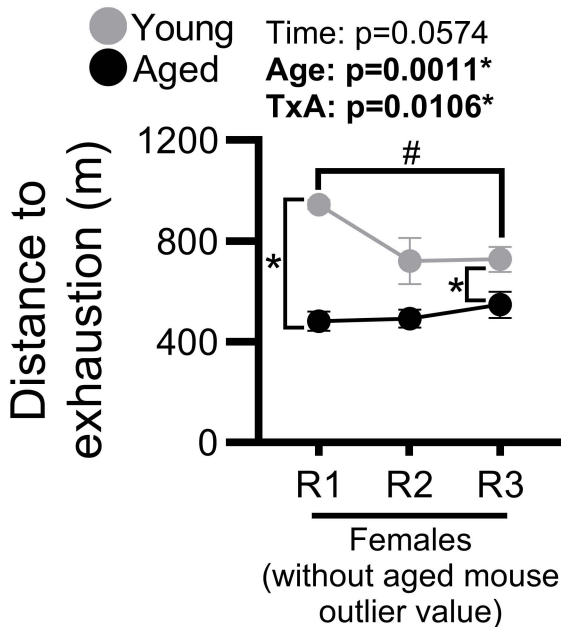

Supplement: Supplementary file 3 — Figure S2: Distance to exhaustion in young (n = 6) and aged (n = 5) female mice with one aged female mouse outlying value omitted was analyzed by 2‐way ANOVA (Time × Age) with Sidak's test used post hoc. #p < 0.05 vs. run 1 in young mice. *p < 0.05 between ages. Data are represented as mean ± SEM. [file ACEL-25-e70573-s001.pdf]

**a**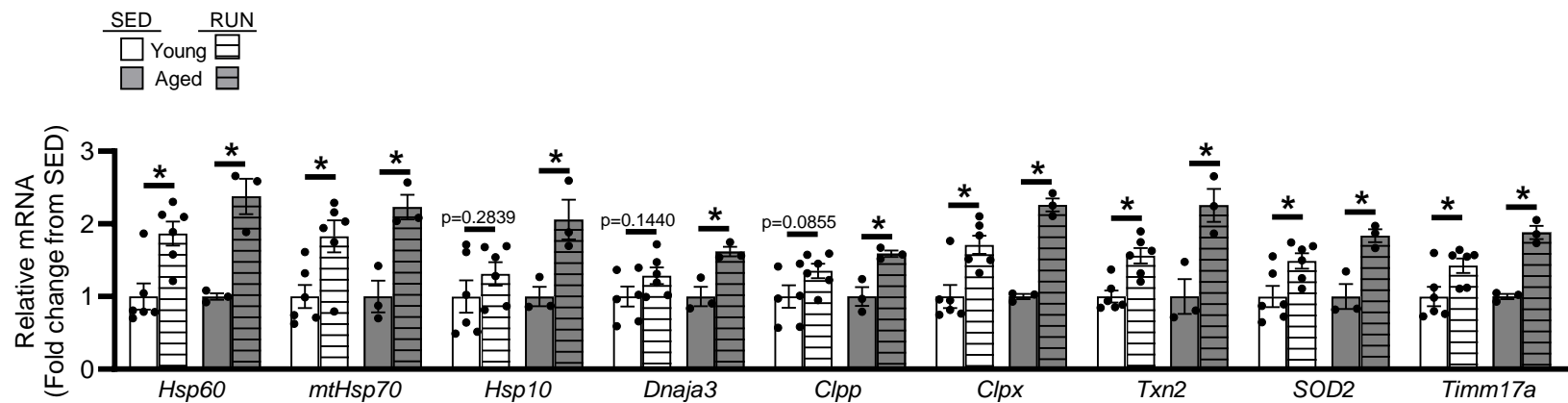**b**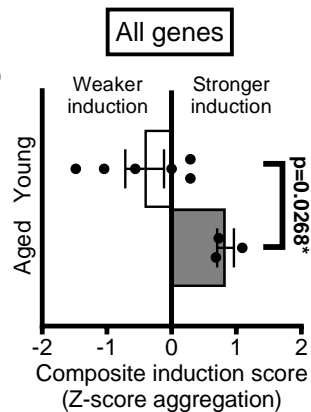

Commonly altered genes only

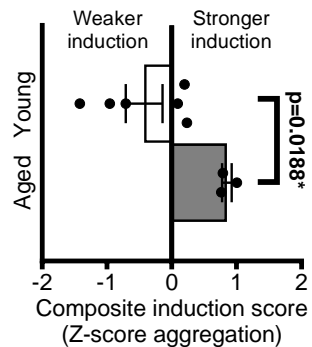

Supplement: Supplementary file 4 — Figure S3: The mtUPR transcriptional response to an acute, unaccustomed bout in young (4‐months; n = 6/group) or aged (24‐months; n = 3/group) male mice assigned to remain sedentary (SED) or undergo 3 days of repetitive physical stress (RUN). (a) mtUPR target genes were assessed by qRT‐PCR in the medial gastrocnemius and (b) composite induction scores for each mouse were calculated using z‐score aggregation to assess the overall magnitude of mtUPR induction using (LEFT) all genes in the panel or (RIGHT) only genes commonly induced in both ages. All data were analyzed using unpaired two‐tailed t‐tests. Data are represented as mean ± SEM. [file ACEL-25-e70573-s004.pdf]

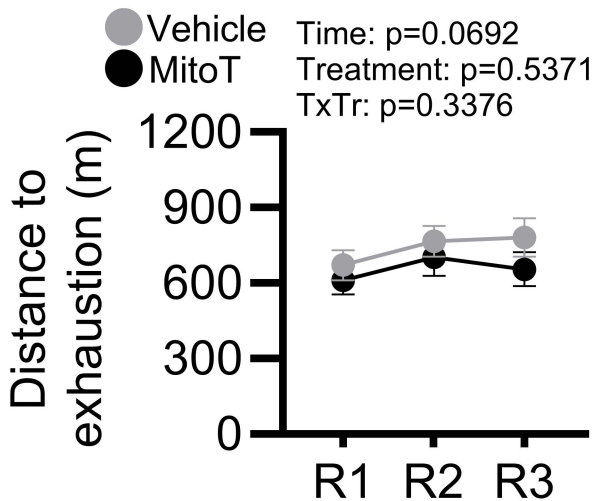

Supplement: Supplementary file 6 — Figure S5: Distance to exhaustion was calculated for each of the 3 consecutive runs to fatigue in mice treated with vehicle or 1 mg/kg MitoTEMPO (MitoT) and analyzed by 2‐way ANOVA (Time × Treatment). Data are represented as mean ± SEM. [file ACEL-25-e70573-s008.pdf]
