# Supplementary material for: Oxidative Stress and Diminished Mitochondrial Proteostatic Reserve Are Linked to Enhanced mtUPR Initiation in Aged Mouse Muscle
Source: Aging Cell. 2026 Jun 4;25(6):e70573. doi: 10.1111/acel.70573 (PMC13238549; doi:10.1111/acel.70573)
Supplement: Supplementary file 5 — Figure S4: HSF1 does not appear to be involved in the mtUPR transcriptional response in aged skeletal muscle at the time points assessed. (a) HSF1 total protein content was measured in the medial gastrocnemius of young (4‐months) and aged male (24‐months) and female (22‐months) mice assigned to remain sedentary (SED; n = 6/age/sex) or undergo 3 days of physical stress (RUN; n = 5 aged males, n = 6/age/sex all other groups) by Western blot and assessed by 2‐way ANOVA (Age × Run). The other gastrocnemius of aged mice was fractionated and the (b) nuclear‐to‐cytosolic subcellular localization of HSF1 was measured by Western blot. (c) Hsf1 mRNA content was measured in the medial gastrocnemius by qRT‐PCR and analyzed by unpaired two tailed t‐tests. Representative Western blot for (a) is shown to the right of the respective bar graph. Data are represented as mean ± SEM. [file ACEL-25-e70573-s006.pdf]

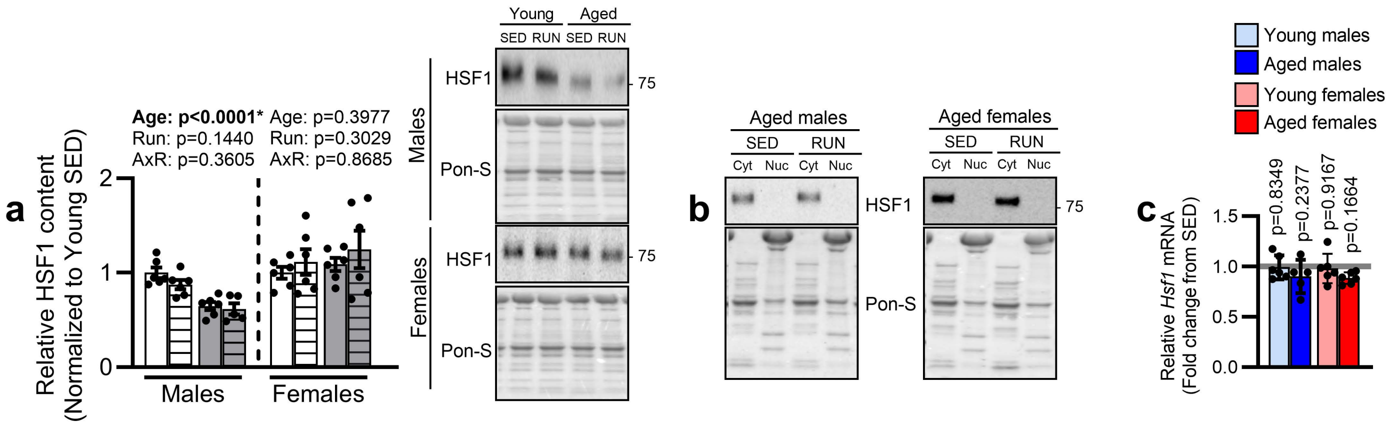

a
Relative HSF1 content (Normalized to Young SED)
Age: p<0.0001*
Run: p=0.1440
AxR: p=0.3605
Age: p=0.3977
Run: p=0.3029
AxR: p=0.8685
Males
Females
Young
Aged
SED RUN SED RUN
HSF1
Pon-S
75
b
Aged males
Aged females
SED
RUN
Cyt Nuc Cyt Nuc
HSF1
Pon-S
75
c
Relative Hsf1 mRNA (Fold change from SED)
p=0.8349
p=0.2377
p=0.9167
p=0.1664
Young males
Aged males
Young females
Aged females
