# Supplementary material for: Oxidative Stress and Diminished Mitochondrial Proteostatic Reserve Are Linked to Enhanced mtUPR Initiation in Aged Mouse Muscle
Source: Aging Cell. 2026 Jun 4;25(6):e70573. doi: 10.1111/acel.70573 (PMC13238549; doi:10.1111/acel.70573)
Supplement: Supplementary file 7 — Table S1: Western blot antibodies. [file ACEL-25-e70573-s009.docx]

**Table S1.** Western blot antibodies

|  | Antibody | Dilution | Host species | Supplier (cat. #) |
| --- | --- | --- | --- | --- |
| 1° Ab | HSP60 | 1:1000 | Rabbit | Cell signaling (12165) |
|  | mtHSP70 | 1:1000 | Rabbit | Cell signaling (3595) |
|  | HSF1 | 1:1000 | Rabbit | Cell signaling (4356) |
|  | LONP1 | 1:1000 | Rabbit | Cell signaling (28020) |
|  | ATF5 | 1:10000 | Rabbit | Invitrogen (MA5-38080) |
|  | ATF4 | 1:500 | Rabbit | Cell signaling (11815) |
|  | CHOP | 1:1000 | Mouse | Cell signaling (2895) |
|  | GAPDH | 1:40000 | Mouse | Santa Cruz (sc-32233) |
|  | Histone H2B | 1:1000 | Rabbit | Cell signaling (12364) |
|  | HSP10 | 1:1000 | Rabbit | Cell signaling (53668) |
|  | Total JNK | 1:1000 | Rabbit | Cell signaling (9252) |
|  | CLPP | 1:100 | Mouse | Santa Cruz (sc-271284) |
|  | OXPHOS cocktail | 1:1000 | Mouse | Invitrogen (45-8099) |
|  | YME1L1 | 1:10000 | Rabbit | Proteintech (11510-1-AP) |
|  | P-JNK (Thr183/Tyr185) | 1:1000 | Rabbit | Cell signaling (9251) |
|  | Total eif2α | 1:1000 | Rabbit | Cell signaling (5324) |
|  | P-eif2α (Ser51) | 1:1000 | Rabbit | Cell signaling (3597) |
|  | P38 MAPK | 1:1000 | Rabbit | Cell signaling (8690) |
|  | p-p38 MAPK (Thr180/Tyr182) | 1:1000 | Rabbit | Cell signaling (4511) |
|  | COX IV | 1:10000 | Rabbit | Cell signaling (4844) |
| 2° Ab | Anti-Rabbit IgG (H+L) | 1:10000 | Goat | Bethyl Laboratories (A-120-101P) |
|  | Anti-Mouse IgG (H+L) | 1:10000 | Goat | Bethyl Laboratories (A-90-116P) |
|  | Anti-Mouse IgG (Heavy chain specific) | 1:10000 | Donkey | Jackson Immunoresearch (115-035-008) |
