# Supplementary material for: Oxidative Stress and Diminished Mitochondrial Proteostatic Reserve Are Linked to Enhanced mtUPR Initiation in Aged Mouse Muscle
Source: Aging Cell. 2026 Jun 4;25(6):e70573. doi: 10.1111/acel.70573 (PMC13238549; doi:10.1111/acel.70573)
Supplement: Supplementary file 8 — Table S2: qRT‐PCR SYBR Green primer sequences. [file ACEL-25-e70573-s007.docx]

**Supplementary Table 2.** qRT-PCR SYBR Green primer sequences

| **Gene** | **Forward (5’-3’)** | **Reverse (5’-3’)** | **Amplicon size (bp)** |
| --- | --- | --- | --- |
| *Rplp0* | CAACCCAGCTCTGGAGAAAC | GTTCTGAGCTGGCACAGTGA | 169 |
| *Atf4* | TCGATGCTCTGTTTCGAATG | GGCAACCTGGTCGACTTTTA | 179 |
| *CHOP* | CCCAGGAAACGAAGAGGAAGAAT | GGCCATAGAACTCTGACTGGAAT | 148 |
| *Hsp60* | GCTAAGAATGCAGGTGTTGAAGG | CTGTAGTTAGCAAGGAGGCCAC | 187 |
| *mtHsp70* | GAAGAAGGAACGTGTTGAAGCAG | CCTGATGTTCTCTCCTGTCTCAC | 181 |
| *Lonp1* | CATTGATGAGGTGGACAAGATTGG | CATTGGCCGTGCAGATGAATAG | 158 |
| *ClpP* | CGCGCTTATGACATATACTCGAG | CCACCTGGGCTGTTGATATACAT | 161 |
| *Hsf1* | TACTTCAAGCACAACAACATGGC | CAAGAAACAAGGATGCTGGAACT | 138 |
| *Clpx* | ACAATGTAGAGAAAGCACAACAAGG | ACTTGAACTGTCTCTCCACGTAG | 196 |
| *Txn2* | CAGTTGTTGTGGACTTTCATGCA | GCTGACACCTCATATTCAATGGC | 157 |
| *SOD2* | CCGAGGAGAAGTACCACGAG | GCTTGATAGCCTCCAGCAAC | 174 |
| *Timm13* | TGCTCCAGAGAATGACGGAC | GGTATTCCAGGCGTCCATGTAG | 125 |
| *Timm17a* | ATCCTGGCAGCAAGAAATGGA | GGCAACTGGGAGTGATCTTCA | 161 |
