# Supplementary material for: Oxidative Stress and Diminished Mitochondrial Proteostatic Reserve Are Linked to Enhanced mtUPR Initiation in Aged Mouse Muscle
Source: Aging Cell. 2026 Jun 4;25(6):e70573. doi: 10.1111/acel.70573 (PMC13238549; doi:10.1111/acel.70573)
Supplement: Supplementary file 9 — Table S3: TaqMan primers. [file ACEL-25-e70573-s005.docx]

**Supplementary Table 3.** TaqMan primers

| **Gene** | **Assay ID** |
| --- | --- |
| *Atf5* | Mm04179654_m1 |
| *Yme1l1* | Mm00496843_m1 |
| *Hsp10* | Mm07295795_g1 |
| *Dnaja3* | Mm07297914_m1 |
